# Design and Synthesis of Pyridyl and 2-Hydroxyphenyl Chalcones with Antitubercular Activity

**DOI:** 10.3390/molecules29194539

**Published:** 2024-09-24

**Authors:** Kelphina Aziafor, Ketan Ruparelia, Brandon Moulds, Mire Zloh, Tanya Parish, Federico Brucoli

**Affiliations:** 1 Leicester School of Pharmacy, De Montfort University, Leicester LE1 9BH, UK; p2621865@alumni365.dmu.ac.uk (K.A.); kruparel@dmu.ac.uk (K.R.); brandon.moulds@dmu.ac.uk (B.M.); 2 Faculty of Pharmacy, University Business Academy, 21000 Novi Sad, Serbia; mire.zloh@faculty-pharmacy.com; 3 UCL School of Pharmacy, University College London, 29/39 Brunswick Square, London WC1N 1AX, UK; 4 Center for Global Infectious Disease Research, Seattle Children’s Research Institute, 307 Westlake Avenue North, Suite 500, Seattle, WA 98102, USA; tanya.parish@seattlechildrens.org

**Keywords:** chalcones, claisen condensation, mycobacterium tuberculosis, breast cancer, protein tyrosine phosphatase B (Mbt PtpB)

## Abstract

A focussed library of pyridyl and 2-hydroxyphenyl chalcones were synthesized and tested for growth inhibitory activity against *Mycobacterium tuberculosis* H37Rv, and normal and cancer breast cell lines. Pyridyl chalcones bearing lipophilic A-ring, e.g., dichloro-phenyl-(**14**), pyrene-1-yl (**20**)- and biphenyl-4-yl (**21**) moieties were found to be the most potent of the series inhibiting the growth of *M. tuberculosis* H37Rv with IC_90_ values ranging from 8.9–28 µM. Aryl chalcones containing a 3-methoxyphenyl A-ring and either *p*-Br-phenyl (**25**) or *p*-Cl-phenyl (**26**) B-rings showed an IC_90_ value of 28 µM. Aryl-chalcones were generally less toxic to HepG2 cells compared to pyridyl-chalcones. Dose-dependent antiproliferative activity against MDA468 cells was observed for trimethoxy-phenyl (**16**) and anthracene-9-yl (**19**) pyridyl-chalcones with IC_50_ values of 0.7 and 0.3 µM, respectively. Docking studies revealed that chalone **20** was predicted to bind to the *M. tuberculosis* protein tyrosine phosphatases B (PtpB) with higher affinity compared to a previously reported PtpB inhibitor.

## 1. Introduction

Tuberculosis (TB) remains one of the deadliest infectious diseases in the world. In 2022, there were an estimated 10.6 million cases of TB, and 1.3 million people died from this disease globally. After years of constant decline, the TB incidence rate (new cases per 100,000 population per year) increased by 3.9% between 2020 and 2022. Further, in 2022 there were about 3.1 million people that were not diagnosed, or not officially reported to health authorities, thus increasing the alarming scale of TB incidence worldwide [1].

The burden of multi-drug (MDR)- and extensively-drug resistant (XDR)-TB is considerable with 410,000 new cases of multidrug and rifampicin-resistant TB being reported in 2022. TB is a major cause of antimicrobial resistance-related deaths [1], and the high incidence of human immunodeficiency viruses (HIV)-TB co-infection poses additional challenges for the control of this disease that nowadays still represents a global health threat. There is an urgent need to identify easily accessible scaffolds with anti-tubercular activity that can be developed into therapeutics to treat TB infections.

Chalcones represent a privileged scaffold in medicinal chemistry, and both naturally occurring and synthetic chalcones have been reported to exert multiple biological functions. Chalcones bear the 1,3-diarylprop-2-en-1-one framework and occur naturally in ferns and higher plants [2,3,4]. These compounds are precursors in the biosynthesis of flavonoids from which they differ due to the absence of a heterocyclic benzopyrone or benzopyran ring system.

**Figure 1:**
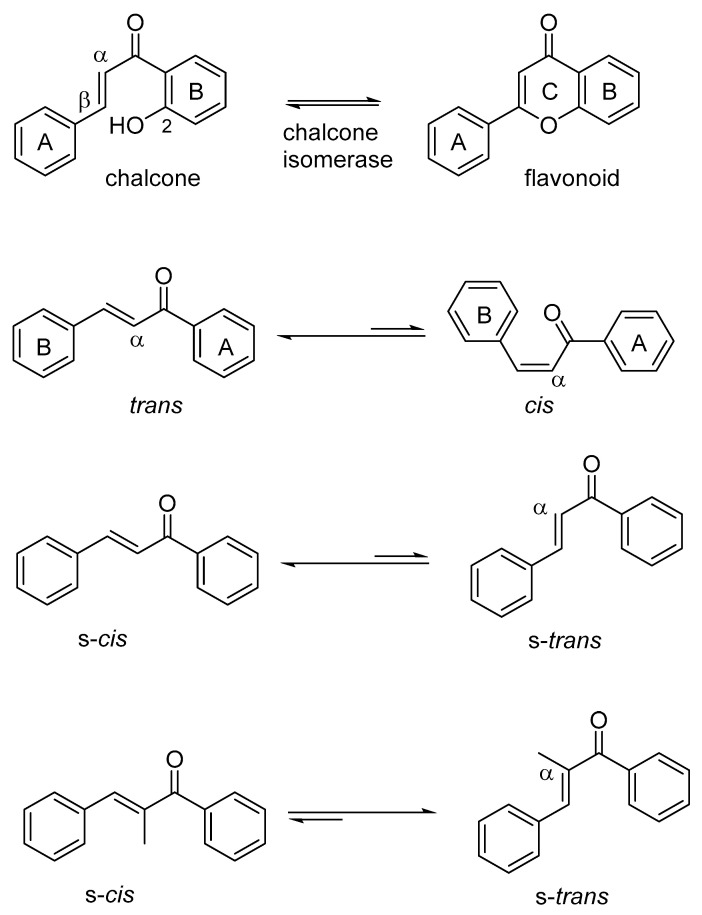
Interconversion of chalcone to flavonoid catalysed by chalcone isomerase. Chalcones *cis*(*Z*)/*trans*(*E*) geometric isomers and *s-cis*/*s-trans* conformers.

The α,β-unsaturated double bond in the enone moiety of chalcones can adopt either *cis*(*Z*) or *trans*(*E*) configuration (Figure 1) [5]. The *trans*-isomer is thermodynamically more stable compared to the *cis*-isomer, and almost all chalcones prepared are isolated in this form. In addition to *cis*/*trans* geometrical isomerism, chalcone can exist in several conformations where the conformers interconvert by rotation along single bonds. Conformational analysis of the orientation of the carbonyl group and the α,β-double bond in chalcones has revealed that in the lowest activation energy they exist as two distinct conformers: the *s-cis,* where the carbonyl and the double bonds are positioned *cis* with respect to each other, or the *s-trans,* where the double bonds are *trans* configured with respect to each other. The *s*-*cis* conformer is reported to be the more stable than the *s*-*trans*, although the latter might predominate when sterically hindered substituents, such as α-methyl groups, are present [6]. Therefore, chalcone conformational equilibria might be influenced by substitution patterns on the aryl rings [7].

**Figure 2:**
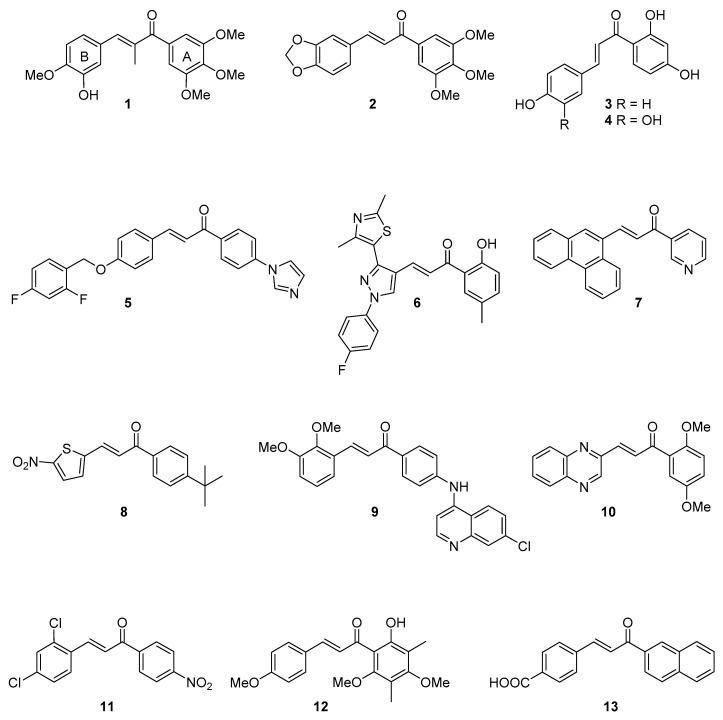
Chemical structures of naturally occurring and synthetic chalcone derivatives possessing anti-tubercular and anti-proliferative activities.

The chalcones frame is very versatile and amenable to multiple synthetic modifications, leading to analogues endowed with remarkable biological properties including anti-inflammatory, anti-proliferative, antiviral, antibacterial, and antifungal activities [8,9,10,11]. Members of this class of compounds displayed significant anti-proliferative activity at nanomolar levels. For example, α-methyl chalcone derivative **1** inhibited the growth of K562 human leukaemia cells [7], whereas our previously reported chalcone anticancer prodrug **2** (DMU-135) halted the proliferation of MDA-468 breast cancer cell lines upon CYP1 bioactivation [12]. Dietary natural chalcones such as isoliquiritigenin **3** (ISL, 4, 2′,4′-trihydroxychalcone) and its tetrahydroxy analogue butein **4** (3,4,2′,4′-tetrahydroxychalcone) have been shown to possess anticancer activities (Figure 2). Isoliquiritigenin has been reported to induce apoptosis in human hepatoma cells, and butein was reported to act as an EGF receptor tyrosine kinase inhibitor in cell-signalling pathways [13,14,15]. It has been suggested that chalcones with phenols in both A- and B-rings (e.g., butein, ISL) are generally more toxic than chalcones that possessed a benzene A-ring and a polyphenol B-ring (pyrogallol, resorcinol) [14].

Chalcone scaffolds have also been employed to design anti-tubercular agents including imidazole derivative **5** and pyrazole-thiazole conjugate **6**, which were found to be active against virulent (H37Rv) and avirulent (ATCC 25177) strains of *M. tuberculosis*, respectively [16,17]. It can be noted that the presence of heterocyclic rings in (or closely linked to) the chalcone frame might lead to enhanced anti-tubercular activity, as can be observed in chalcone hybrids containing either pyridine (i.e., **7**) [18,19], nitro-thiophene (i.e., **8**) [20], quinoline (i.e., **9**) [21,22], and quinoxaline (i.e., **10**) rings. Moreover, the introduction of a nitro group (i.e., **11**) or hydroxy/poly-methoxy functional groups (i.e., **12**) in chalcone A-rings resulted in derivatives active against *M. tuberculosis* H37Rv [23,24].

Interestingly, naphthylchalcone analogues were found to inhibit *M. tuberculosis* protein tyrosine phosphatases A and B (Mtb PtpA and B) with IC_50_ values in the low-micromolar range. Docking studies revealed that the presence of the bulky, hydrophobic 2-naphthyl substituent in the A-ring of chalcone **13** played an important role in the inhibition of Mtb PtpB [22].

Given the therapeutic potential of this class of compounds, we sought to investigate a series of aryl- and heteroaryl-chalcones for their antitubercular activity and cytotoxicity in cancer and non-cancer breast cells, and HepG2 cells. The structural insights and SARs reported here may be beneficial for the design of potent and cost-effective anti-tubercular agents.

## 2. Results and Discussion

### 2.1. Chemistry

Two chalcone frameworks were selected for our synthetic campaign and included the (*E*)-1-(phenyl-1-(pyridin-4-yl)prop-2-en-1-one (Scaffold A or pyridyl chalcones) and (*E*)-1-(4-chloro/bromophenyl)- or (*E*)-3-(1,3-diphenyl-1*H*-pyrazol-4-yl)-1-(hydroxy/methoxy-phenyl)prop-2-en-1-one (Scaffold B or aryl chalcones), as previously reported derivatives bearing pyridinyl and hydroxyphenyl moieties (e.g., compounds **6** and **7**) exhibited attractive antitubercular activities.

**Scheme 1:**
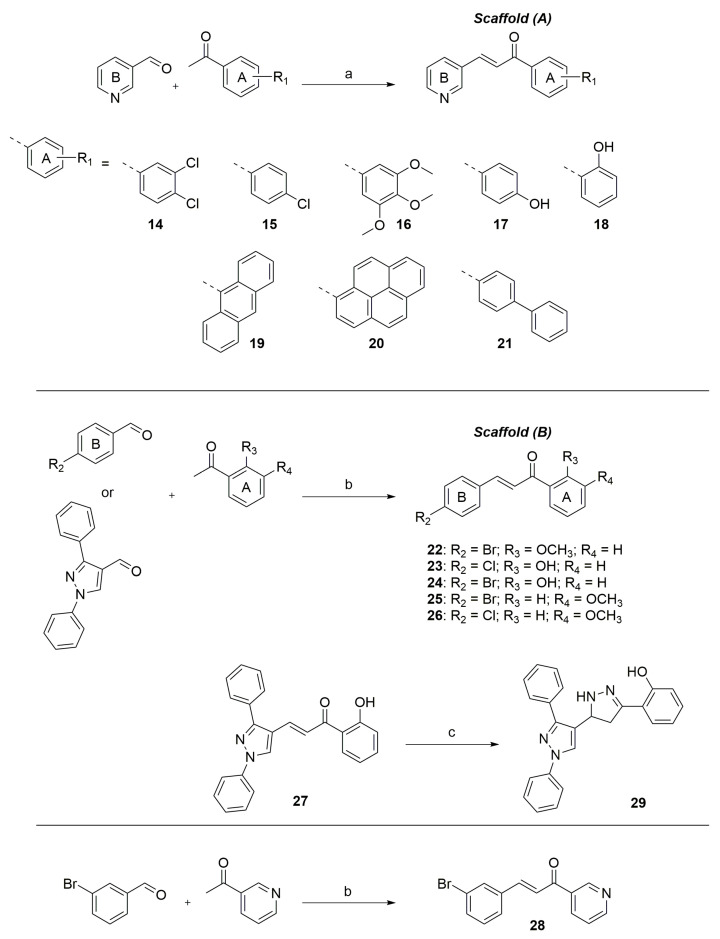
Synthesis of pyridyl and aryl-chalcones used in this study. Reagents and conditions: (a) LDA, dry THF, −78 °C then RT. (b) NaOH (aq), MeOH, 0 °C. (c) NH_2_NH_2_·H_2_O, EtOH, RT, overnight.

Chalcones are readily synthesized by the Claisen–Schmidt condensation reaction of a benzaldehyde and acetophenone in equimolar quantities in the presence of a base or acid catalyst. Synthesis of Scaffold A required the use of aprotic anhydrous solvent with freshly made lithium diisopropylamide (LDA) as the base, whereas chalcones containing Scaffold B were prepared employing aqueous sodium hydroxide solution (50% *w*/*v*) with methanol as the solvent (Scheme 1). A series of acetophenones with different physicochemical properties were incorporated in Scaffold A analogues to assess their ability to penetrate the thick lipophilic cell envelope of *M. tuberculosis* and arrest its growth. Three previously reported chalcones (**2**, **20** and **21**), [25,26,27] which were designed as anti-proliferative and CYP1 enzyme inhibiting agents, were re-synthesised and evaluated, for the first time, against *M. tuberculosis* H37Rv. Further, to assess the reactivity of the chalcones, we converted **23**, **24** and **27** into pyrazoline derivatives **30**, **31** and **29**, respectively, using hydrazine hydrate, and evaluated their anti-tubercular activity determining the inhibitory concentration of the tricyclic compounds.

### 2.2. Biological Evaluation and Structure Activity Relationship Elucidation

The compounds were screened against wild-type *M. tuberculosis* H37Rv, and inhibitory concentrations were determined. Cytotoxicity was evaluated in HepG2, “normal” MCF10A and breast cancer MDA468 cell lines (Table 1). Pyridyl chalcones **14**, **20** and **21** exhibited anti-tubercular activity in the low micromolar range with IC_90_ values ranging from 8.9–28 µM. The biphenyl-moiety bearing chalcone **21** [25] was the most active against *M. tuberculosis* with an IC_90_ value of 8.9 µM, although it showed toxicity across the panel of “normal” and cancer eukaryotic cells used in this study. This trend was observed for all Scaffold (A) chalcones excluding pyridyl chalcones **17** and **18** that contained hydroxyphenyl units in their A rings. The presence of hydroxy groups in the A rings of **17** and **18** abolished the chalcones anti-tubercular activity, while preserving their ability to inhibit the growth of MDA-468 cancer cells with IC_50_ values of 30 and 8 µM, respectively. Trimethoxy-phenyl (**16**) and anthracene-9-yl (**19**)-including chalcones exhibited IC_90_ values of 59 and 48 µM, respectively, against *M. tuberculosis*, and were the most cytotoxic, amongst the compounds tested, in MDA468 cells with IC_50_ values of 0.7 and 0.3 µM, respectively. HepG2 toxicity was observed for the majority of pyridyl-chalcones.

The contribution of the B ring to the anti-tubercular activity of Scaffold A derivatives was also evaluated. To this end, the “reversed” chalcone **28** was synthesised to establish whether the replacement of the pyridine B ring with a *m*-bromo-phenyl moiety affected the anti-tuberculosis activity. It was found that the position of the pyridine unit within the chalcone frame, i.e., ring A or B, did not overly influence the *M. tuberculosis* growth inhibitory properties of **28** that were comparable to those of other pyridyl chalcones, such as **15**, **16**, and **19** synthesised in this study. Chalcone **28** exhibited cytotoxic activity in MCF10A and MDA468 cell lines. Further, we tested our previously reported chalcone **2**, [12], which included the 3,4-methylenedioxy phenyl B ring. Chalcone **2**, which contains a 3,4-methylenedioxyphenyl ring B, did not exhibit anti-tubercular activity but showed selectivity for CYP1-expressing MDA468 breast cancer cells.

Subsequent steps in the chalcone frame modification included the replacement of pyridyl B ring with either *p*-Cl-/*p*-Br-phenyl or diphenyl-pyrazole rings leading to compounds **22**–**27**.

Scaffold B chalcones bearing a 2-hydroxyphenyl units in ring A were inactive, i.e., **23**, **24**, and **27** (IC_90_ = 97–100 µM), whereas 3-methoxyphenyl moieties were well tolerated with analogues **25** and **26** showing IC_90_ values of 29 and 28 µM, respectively. Interestingly, chalcone **22**, which had a 2-methoxyphenyl unit in ring A, showed very weak activity against *M. tuberculosis* with an IC_90_ value of 59 µM.

**Figure 3:**
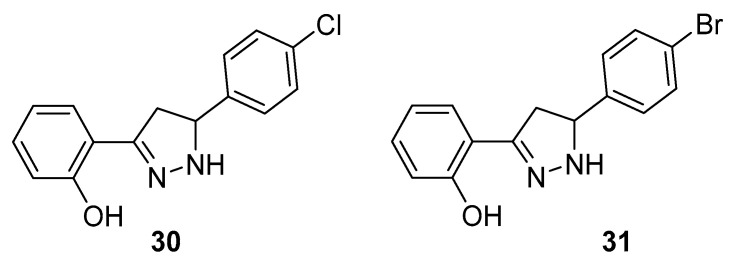
Structure of previously reported mycobactin analogues **30** and **31** synthesized in this work for comparative purposes.

At this stage, we were interested to determine whether modification of the framework of chalcones bearing a 2-hydroxyphenyl A ring might lead to derivatives with improved anti-tubercular activities. To this end, we converted the inactive chalcone **27** into pyrazoline **29**, which was found to exhibit an IC_90_ value of 25 µM (IC_50_ = 11 µM). This growth-inhibitory activity correlated well with the anti-tubercular properties of previously reported mycobactin analogue-pyrazolines **30** and **31** (IC_90_ = 34 µM; IC_50_ = 23 µM), [28], which were re-synthesised and screened in this study (Figure 3).

### 2.3. Molecular Modelling

Given the structural similarity of known Mtb PtpB inhibitor **13** with pyridyl chalcones **19**, **20**, and **21**, which contained lipophilic A-rings, we sought to predict whether these analogues would bind to either Mtb PtpA (PDB ID: 1U2P) or Mbt PtpB (PDB ID: 2OZ5). CYP1 inhibitor **2** (DMU-135) was also included in the phosphatases docking studies.

Two different software packages were used to study putative binding of these molecules in both enzymes, i.e., AutoDock Vina v.1.1.2 [29] and GOLD v. 2024.1.0 [30], according to our previous methods [31] (Table 2). The docking score is an estimation of the binding affinity between the small molecules and the target proteins, with more negative values indicating stronger predicted binding, while higher GoldScore fitness values generally indicate a stronger predicted binding affinity between the molecule and the target protein. It is important to note that docking studies are primarily used for relative comparisons of binding affinities between different small molecules for a specific protein target, rather than direct comparisons of binding across different protein targets. Nevertheless, the consistent trend observed in both the Vina docking scores and GoldScore fitness values suggested that the chalcones analysed in this modelling study (**2**, **13**, **19**, **20**, and **21**) may exhibit a higher binding affinity towards Mtb PtpB (PDB ID: 2OZ5) compared to Mtb PtpA (PDB ID: 1U2P). Compound **20**, which exhibited an IC_50 H37Rv_ of 8.8 µM, was predicted to be the most promising candidate, showing the best docking scores, when compared to **13**, against PtpB, according to the Vina docking program. Based on the GoldScore fitness scores, compound **19** appeared to be a stronger binder than **20**, albeit marginally. Chalcone **2**, which had no antitubercular activity, showed the least favourable docking score for both PtpA or PtpB amongst the compounds analysed despite having a favourable predicted binding by GOLD. Although it was found in the literature that DMU-135 (**2**) possessed very low PtpA inhibition activity [32], our prediction using GOLD showed that this chalcone would not be involved with the important residues of the binding site, and therefore cannot be considered as a potentially active molecule. Additionally, the AutoDock Vina algorithm better dealt with the absence of the second inhibitor copy in the binding site, as explained in the Methods section and shown in Table 3.

Previous virtual screening for an inhibitor of Mbt PtpB target employed a consensus scoring approach by comparing the results of five different software packages and revealed the importance of hydrogen bonds formed between the ligand and three residues (K164, D165, and R166) and a lipophilic pocket formed by P81, H94, F98, L101, Y125, M126, F161, L199, I203, L227, V231, and L232 [33].

**Figure 4:**
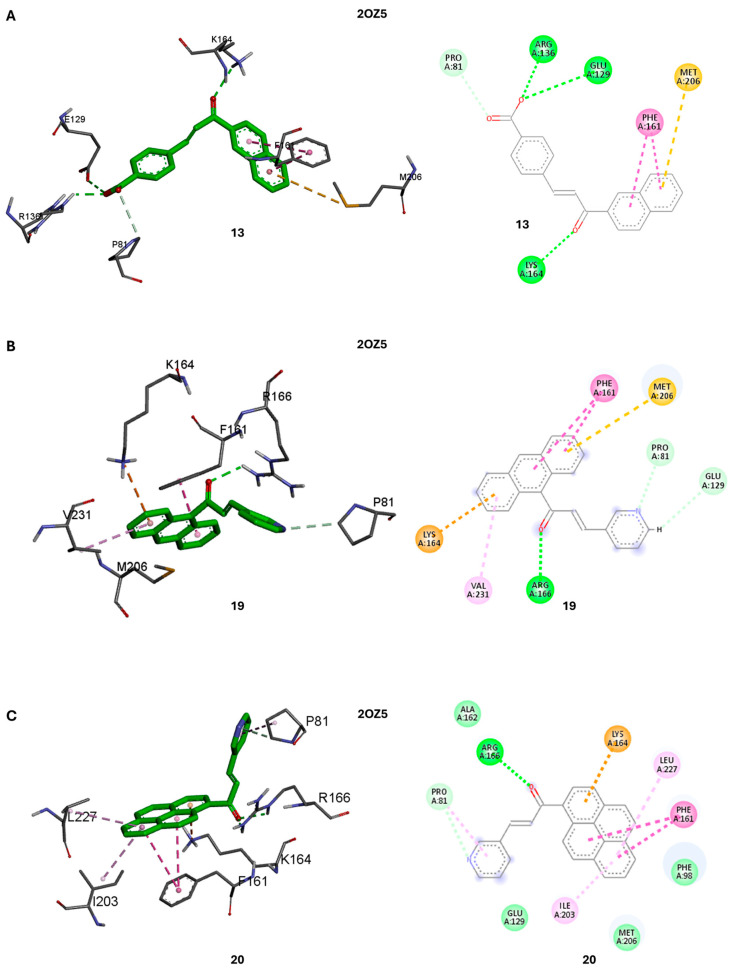
Key interactions between (**A**) **19**, (**B**) **20,** and (**C**) **13**, and *M. tuberculosis* PtpB (PDB ID: 2OZ5). The three-dimensional representations of the ligands with the carbon atoms coloured in green interacting with the binding site residues are shown on the left side, while the two-dimensional ligand plot interactions are shown on the right side for each complex. The dotted lines indicate the interactions, with green coloured lines indicating conventional hydrogen bonds, purple lines depicting π-π interactions, pink lines indicating π-hydrophobic interactions and orange lines depicting π-sulfur interactions.

A detailed analysis of the most favourable binding poses of **13** in PtpB (2OZ5) obtained by AutoDock Vina showed the presence of a hydrogen bond between K164 with the keto group of the chalcone frame (Figure 4A). For **19**, only one of three hydrogen bonds (E129, K164, and R166) specified in the model is detected in the binding pose, e.g., R166 side chain interacting with keto group of **19**. However, additional relevant interactions are observed between the anthracenyl unit of **19** and F161, K164, M206, and V231 residues, and between the pyridine ring of **19** and P81 and E129 of PtpB (2OZ5) (Figure 4B).

Similarly, in the binding pose of **20**, only one hydrogen bond was detected with R166, although the molecule interacted with an even higher number of relevant residues, namely P81 (with pyridine ring of **20**), F98, F161, K164, I203, and L227 (with pyrene ring of **20**), compared to both **13** and **19** (Figure 4C). This likely led to the more favourable (Vina) docking score of **20** (−9.6 kcal/mol) compared to **13** (−9.2 kcal/mol) for the PtpB (PDB ID: 2OZ5) target (Table 2).

While these computational findings provide valuable guidance for prioritising compounds and targets for further experimental evaluation, it is crucial to validate these predictions through biochemical and biophysical assays to confirm the actual binding affinities and selectivity profiles of these compounds against Mtb PtpA and PtpB.

## 3. Experimental

### 3.1. General Chemistry Information

Chemicals and reagents were purchased from Fisher Scientific, Loughborough, UK, Merck Life Science UK Limited, Gillingham, UK, and VWR INTERNATIONAL Ltd., Lutterworth, UK, and used as received. ^1^H and ^13^C Nuclear Magnetic Resonance (NMR) spectroscopy analyses were carried out using a JEOL ECZR 600 MHz (equipped with a ROYAL probe). Solvent signals for hydrogen and carbon NMR were used as the internal reference. Chemical shifts (*δ*_H_) are quoted in parts per million and are relative to the solvents’ residual peaks in the ^1^H and ^13^C NMR spectra: CDCl_3_ (7.26 and 77.0 ppm), MeOD-*d*_4_ (3.31 and 49.1 ppm), and DMSO-*d*_6_ (2.50 and 39.52 ppm). Coupling constants (J) are given in Hertz (Hz), and the signal multiplicity is described as singlet (s), doublet (d), doublet of doublets (dd), triplet of doublets (td), triplet (t), quartet (q), and multiplet (m). The deuterated solvents (CDCl_3_, DMSO-*d*_6,_ and MeOD-*d*_4_) used for NMR spectroscopy experiments were purchased from Cambridge Isotope Laboratories Inc. (Tewksbury, MA, USA). Thin Layer Chromatography (TLC) was performed using aluminium backed 20 × 20 cm silica gel 60 F_254_ plates, which were purchased from Merck (Rahway, NJ, USA) for viewing colourless spots under 254 nm wavelength ultraviolet light. Flash column chromatography purifications of the intermediates and final products were conducted in a glass column using irregular, 60 Å pore size silica gel, 63–200 μm, 70–230 mesh. LC-MS analysis was conducted on an Agilent 1260 LC coupled with 6120 series Quadrupole LC-MS system with a G1311B quaternary pump. The column used was a Phenomenex Luna C18(2) 4.6 × 150 mm 5 microns). HRMS analysis was performed on a Waters UPLC coupled with a Xevo G2-XS QTOF (Waters, Wilmslow, UK). Chromatographic analysis was achieved using an Acquity BEH C18 UPLC column (1.7 µm, 2.1 × 50 mm) at 40 °C. The mobile phases consisted of 0.1% formic acid in LCMS-grade water (A) and 0.1% formic acid in LCMS-grade acetonitrile (B). The flow rate was 0.5 mL min^−1^, with the following gradient program: 0 min 5% B, 0–0.5 min 5% B, 0.5–1.5 min 5–25% B, 1.5–2 min 25% B, 2–3.5 min 25–70% B, 3.5–3.9 min 70% B, 3.9–5 min 5% B. The total run time was 5 min. The injection volume was 0.5 µL. Mass spectrometry analysis was performed in positive ion mode using an ESI source using a scan range of 50–1000 Da at 0.2 scans s^−1^. Positive ion electrospray was used with a capillary voltage of 1 kV; cone voltage 20 V; source offset 80 V; source temperature 120 °C; desolvation gas temperature 450 °C; cone gas 1000 L/hr. Leucine enkephalin (200 ng/mL) was used as a reference mass (*m*/*z* 556.2771) at 10 µL min^−1^; scan time 0.3 s; scan interval 40 s. Data was processed using MassLynx 4.2 software (Waters, Wilmslow, UK).

#### 3.1.1. General Method for Scaffold A or Pyridyl Chalcones Synthesis

Compounds **14**–**21**, and **28**. A solution of *n*-butyl lithium (1.6 M in hexane, 1 equiv.) was added dropwise to a stirred solution of *N*,*N*-diisopropylethylamine (1 equiv.) in anhydrous THF (10 mL) at −78 °C under nitrogen. After 30 min, a solution of the appropriate acetophenone (1 equiv.) in anhydrous THF (5 mL) was added to the solution at −78 °C. Subsequently, after 10 min, a solution of the pyridine-3-carbaldehyde (1 equiv.) in THF (5 mL) was added to the reaction mixture, which was allowed to reach room temperature and stirred overnight. The mixture was quenched with water (20 mL), neutralised with 1 N hydrochloric acid, and extracted with ethyl acetate (3 × 50 mL). The combined organic fractions were washed with brine (20 mL), dried over MgSO_4_, and the solvent was removed in vacuo. The final compounds were purified by flash column chromatography on silica gel using a gradient elution with hexane: ethyl acetate: triethylamine (10–80% ethyl acetate, 5% triethylamine).

(*E*)-1-(3,4-dichlorophenyl)-3-(pyridin-3-yl)prop-2-en-1-one (**14**). An off-white solid. Yield: 67%. ^1^H-NMR (600 MHz, DMSO-*d*_6_) δ 9.04 (s, 1H), 8.62–8.41 (m, 3H), 8.11 (s, 2H), 7.82 (d, J = 40.8 Hz, 2H), 7.54 (s, 1H). ^13^C-NMR (151 MHz, DMSO-*d*_6_) δ 181.7, 151.3, 150.6, 148.5, 141.7, 137.4, 135.4, 131.2, 130.5, 130.3, 128.6, 123.9, 123.2. ESI-MS [M + H]. Calculated for C_14_H_9_Cl_2_NO: 277.0; Observed: 278.0. HRMS *m*/*z* calculated 278.0139 [M + H]^+^, found 278.0138 [M + H]^+^.

(*E*)-1-(4-chlorophenyl)-3-(pyridin-3-yl)prop-2-en-1-one (**15**). An off-white solid. Yield 55%. ^1^H-NMR (600 MHz, DMSO-*d*_6_) δ 8.96 (d, J = 2.1 Hz, 1H), 8.58 (dd, J = 4.8, 1.5 Hz, 1H), 8.31 (dt, J = 8.0, 1.9 Hz, 1H), 7.94 (d, J = 15.8 Hz, 1H), 7.71 (d, J = 15.6 Hz, 1H), 7.62 (d, J = 7.7 Hz, 1H), 7.47 (dd, J = 8.0, 4.7 Hz, 1H), 7.44 (t, J = 2.1 Hz, 1H), 7.37 (t, J = 7.9 Hz, 1H), 7.02–7.09 (1H). ^13^C-NMR (151 MHz, DMSO-*d*_6_) δ 187.7, 150.3, 149.6, 144.5, 143.3, 139.4, 135.4, 131.2, 130.5, 130.3, 128.6, 124.9, 123.1. ESI-MS [M + H]: Calculated for C_14_H_10_ClNO: 243.1; Observed: 244.1. HRMS *m*/*z* calculated 244.0529 [M + H]^+^, found 278.0138 [M + 2H_2_O + H]^+^.

(*E*)-3-(pyridin-3-yl)-1-(3,4,5-trimethoxyphenyl)prop-2-en-1-one (**16**). A pale-yellow powder. Yield 65%. 1H-NMR (600 MHz, CDCl_3_) δ 8.86 (d, J = 2.1 Hz, 1H), 8.62 (dd, J = 4.8, 1.5 Hz, 1H), 7.93 (dt, J = 7.9, 1.9 Hz, 1H), 7.78 (d, J = 15.6 Hz, 1H), 7.53 (d, J = 15.8 Hz, 1H), 7.35 (dd, J = 7.9, 4.8 Hz, 1H), 7.24 (s, 1H), 3.93 (d, J = 5.3 Hz, 9H). ^13^C-NMR (151 MHz, CDCl_3_) δ 188.5, 153.2, 151.1, 149.9, 142.9, 140.8, 134.7, 133.0, 130.7, 123.8, 123.6, 106.2, 56.4. ESI-MS [M + H]: Calculated for C_17_H_17_NO_4_: 299.1; Observed: 300.1. HRMS *m*/*z* calculated 300.1236 [M + H]^+^, found 300.1230 [M + H]^+^.

(*E*)-1-(2-hydroxyphenyl)-3-(pyridin-3-yl)prop-2-en-1-one (**18**). An off-white powder. Yield 58%. ^1^H-NMR (600 MHz, CDCl_3_) δ 8.89 (d, J = 2.2 Hz, 1H), 8.66 (dd, J = 4.8, 1.5 Hz, 1H), 7.97 (dt, J = 7.9, 1.7 Hz, 1H), 7.92–7.89 (m, 2H), 7.73 (d, = 15.6 Hz, 1H), 7.54–7.52 (m, 1H), 7.39 (dd, J = 7.9, 4.8 Hz, 1H), 7.05 (dd, J = 8.4, 1.0 Hz, 1H), 6.98–6.96 (m, 1H). ^13^C-NMR (151 MHz, CDCl_3_) δ 193.1, 163.7, 151.4, 150.1, 141.6, 136.7, 134.7, 130.4, 129.6, 123.8, 122.1, 119.8, 119.0, 118.8, 77.2, 77.0, 76.8. ESI-MS [M + H]: Calculated for C_14_H_11_NO_2_: 225.1; Observed: 226.1. HRMS *m*/*z* calculated 226.0868 [M + H]^+^, found 226.0872 [M + H]^+^.

(*E*)-1-(pyren-1-yl)-3-(pyridin-3-yl)prop-2-en-1-one (**20**). An off-white powder. Yield 64%. ^1^H-NMR (600 MHz, CDCl_3_) δ 8.81 (d, J = 2.1 Hz, 1H), 8.66 (d, J = 9.1 Hz, 1H), 8.63 (dd, J = 4.8, 1.5 Hz, 1H), 8.29–8.26 (m, 3H), 8.24 (d, J = 7.9 Hz, 1H), 8.20 (dd, J = 9.1, 6.0 Hz, 2H), 8.12 (d, J = 8.9 Hz, 1H), 8.08 (t, J = 7.6 Hz, 1H), 7.94 (dt, J = 8.0, 1.9 Hz, 1H), 7.66 (d, J = 16.2 Hz, 1H), 7.55 (d, J = 16.2 Hz, 1H), 7.36 (dd, J = 8.1, 4.8 Hz, 1H). ^13^C-NMR (151 MHz, DMSO-*d*_6_) δ 194.1, 151.1, 150.4, 141.6, 135.1, 133.0, 132.8, 130.7, 130.4, 130.0, 129.4, 129.2, 128.8, 128.7, 127.2, 127.0, 126.8, 126.5, 126.1, 124.4, 124.3, 124.0, 123.9, 123.5. ESI-MS [M + H]: Calculated for C_24_H_15_NO: 333.1; Observed: 334.1. HRMS *m*/*z* calculated 334.1232 [M + H]^+^, found 334.1229 [M + H]^+^.

(*E*)-1-([1,1′-biphenyl]-4-yl)-3-(pyridin-3-yl)prop-2-en-1-one (**21**). A yellow solid. Yield 34%. ^1^H-NMR (600 MHz, CDCl_3_) δ 8.89 (d, J = 1.9 Hz, 1H), 8.64 (dd, J = 4.7, 1.5 Hz, 1H), 8.12 (d, J = 6.5 Hz, 2H), 7.97 (dt, J = 7.9, 1.8 Hz, 1H), 7.83 (d, J = 15.8 Hz, 1H), 7.75 (d, J = 8.6 Hz, 2H), 7.67–7.64 (m, 3H), 7.49 (t, J = 7.6 Hz, 2H), 7.43–7.40 (m, 1H), 7.38 (dd, J = 7.9, 4.8 Hz, 1H). ^13^C-NMR (151 MHz, DMSO-*d*_6_) δ 188.4, 151.0, 150.3, 144.7, 140.5, 138.8, 136.0, 135.1, 130.5, 128.4, 123.9, 123.8. ESI-MS [M + H]: Calculated for C_20_H_15_NO: 285.1; Observed: 286.1. HRMS *m*/*z* calculated 286.1232 [M + H]^+^, found 286.1229 [M + H]^+^.

(*E*)-3-(3-bromophenyl)-1-(pyridin-3-yl)prop-2-en-1-one (**28**). A pale-yellow solid. Yield 55%. ^1^H-NMR (600 MHz, CDCl_3_) δ 9.24 (q, J = 1.0 Hz, 1H), 8.82 (dd, J = 4.8, 1.7 Hz, 1H), 8.29 (ddd, J = 7.9, 2.2, 1.8 Hz, 1H), 7.81 (t, J = 1.7 Hz, 1H), 7.76 (d, J = 15.8 Hz, 1H), 7.57 (dd, J = 7.9, 1.9 Hz, 2H), 7.49–7.46 (m, 2H), 7.32 (t, J = 7.8 Hz, 1H). ^13^C-NMR (151 MHz, CDCl_3_) δ 188.7, 153.4, 149.8, 144.1, 136.6, 135.9, 133.7, 133.2, 131.1, 130.6, 127.3, 123.7, 123.2, 122.5. ESI-MS [M + H]: Calculated for C_14_H_10_BrNO: 286.9; Observed: 287.9. HRMS *m*/*z* calculated 288.0024 [M + H]^+^, found 288.0022 [M + H]^+^.

#### 3.1.2. General Method for Scaffold B Chalcones Synthesis

Compounds **23**, **24**, and **27**. To a solution of the appropriate 2-hydroxyacetophenone (1 equiv.) and aromatic aldehyde (1 equiv.) in MeOH (20 mL) was added NaOH (50% *w*/*v*, 10 equiv.) dropwise under cooling (0–5 °C) and stirring. After the addition was complete the reaction mixture was allowed to reach room temperature and stirred for the required amount of time until disappearance of the starting materials and formation of a precipitate, as confirmed by TLC analysis (i.e., 24 h to 48 h). The solution was then acidified to pH 2 with 2 N HCl, and a yellow-reddish precipitate was collected by filtration. Recrystallisation from EtOH afforded the products.

(*E*)-3-(4-chlorophenyl)-1-(2-hydroxyphenyl)prop-2-en-1-one (**23**). ^1^H-NMR (600 MHz, DMSO-*d*_6_) δ 8.15–8.21 (1H), 7.95–8.02 (1H), 7.87–7.93 (2H), 7.75–7.82 (1H), 7.57–7.54 (m, 1H), 7.51 (dt, J = 9.0, 2.2 Hz, 2H), 7.01–6.98 (m, 2H). ^13^C-NMR (151 MHz, DMSO-*d*_6_) δ 193.7, 161.9, 143.5, 136.7, 135.7, 133.6, 131.1, 131.0, 129.3, 122.8, 121.0, 119.6, 118.0. ESI-MS [M + H]: Calculated for C_15_H_11_ClO_2_: 258.1; Observed: 259.1. HRMS *m*/*z* calculated 259.0526 [M + H]^+^, found 259.0528 [M + H]^+^.

(*E*)-3-(4-bromophenyl)-1-(2-hydroxyphenyl)prop-2-en-1-one (**24**). ^1^H-NMR (600 MHz, DMSO-*d*_6_) δ 12.38 (s, 1H), 8.17 (dd, J = 8.0, 1.7 Hz, 1H), 8.00 (d, J = 15.6 Hz, 1H), 7.83 (d, J = 8.5 Hz, 2H), 7.76 (d, J = 15.6 Hz, 1H), 7.68 (d, J = 8.5 Hz, 1H), 7.65 (d, J = 8.5 Hz, 2H), 7.57–7.54 (m, 1H), 6.99–6.98 (m, 1H). ^13^C-NMR (151 MHz, DMSO-*d*_6_) δ 193.8, 161.9, 143.6, 136.7, 133.9, 132.2, 131.9, 131.5, 131.2, 131.1, 124.6, 122.9, 121.0, 119.6, 118.0. ESI-MS [M + H]: Calculated for C_15_H_11_BrO_2_: 301.9; Observed: 302.9. HRMS *m*/*z* calculated 303.0021 [M + H]^+^, found 303.0024 [M + H]^+^.

(*E*)-3-(1,3-diphenyl-1H-pyrazol-4-yl)-1-(2-hydroxyphenyl)prop-2-en-1-one (**27**). ^1^H-NMR (600 MHz, CDCl_3_) δ 8.38 (s, 1H), 8.00 (d, J = 15.8 Hz, 1H), 7.82 (dd, J = 8.7, 1.1 Hz, 2H), 7.76 (dd, J = 8.1, 1.5 Hz, 1H), 7.73–7.71 (m, 2H), 7.54–7.46 (m, 7H), 7.39–7.36 (m, 1H), 7.02 (dd, J = 8.2, 1.0 Hz, 1H), 6.92–6.89 (m, 1H). ^13^C-NMR (151 MHz, CDCl_3_) δ 193.3, 163.6, 154.1, 139.4, 136.2, 136.1, 132.2, 129.6, 129.4, 128.9, 128.8, 127.4, 127.1, 120.0, 119.4, 119.4, 118.7, 118.6, 118.2. ESI-MS [M + H]: Calculated for C_24_H_18_N_2_O_2_: 366.1; Observed: 367.1. HRMS *m*/*z* calculated 367.1447 [M + H]^+^, found 367.1441 [M + H]^+^.

Compounds **25** and **26**. To a solution of the appropriate methoxy-acetophenone (1 equiv.) and aromatic aldehyde (1 equiv.) in MeOH (20 mL) was added NaOH (50% *w*/*v*, 10 equiv.) dropwise under cooling (0–5 °C) and stirring. After the addition was complete, the reaction mixture was allowed to reach room temperature and stirred for the required amount of time (i.e., 24 h to 48 h). Upon completion, as indicated by the formation of a precipitate and confirmed by TLC, the reaction was quenched with water (30 mL). The resulting mixture was extracted with ethyl acetate (3 × 30 mL). The combined organic extracts were washed with brine (50 mL), dried (MgSO_4_), and the solvent removed under vacuum. The crude product was purified by flash chromatography on silica gel using a gradient elution with hexane: ethyl acetate (30–50% ethyl acetate).

(*E*)-3-(4-bromophenyl)-1-(3-methoxyphenyl)prop-2-en-1-one (**25**). A beige solid. Yield 75%. ^1^H-NMR (600 MHz, DMSO-*d*_6_) δ 7.94 (d, J = 15.6 Hz, 1H), 7.86 (d, J = 8.5 Hz, 2H), 7.76 (d, J = 7.7 Hz, 1H), 7.70 (d, J = 15.6 Hz, 1H), 7.65 (d, J = 8.5 Hz, 2H), 7.60 (t, J = 2.0 Hz, 1H), 7.48 (t, J = 7.9 Hz, 1H), 7.24 (dd, J = 8.2, 2.7 Hz, 1H), 3.84 (s, 3H). ^13^C-NMR (151 MHz, DMSO-*d*_6_) δ 188.9, 159.6, 142.7, 138.9, 134.0, 131.9, 131.2, 131.2, 130.9, 130.0, 124.0, 122.9, 121.1, 119.3, 113.1, 55.4. ESI-MS [M + H]: Calculated for C_16_H_13_BrO_2_: 316.0; Observed: 317.0. HRMS *m*/*z* calculated 317.0177 [M + H]^+^, found 317.0175 [M + H]^+^.

(*E*)-3-(4-chlorophenyl)-1-(3-methoxyphenyl)prop-2-en-1-one (**26**). A pale-yellow solid. Yield 71%. ^1^H-NMR (600 MHz, DMSO-*d*_6_) δ 7.95 (d, J = 4.0 Hz, 2H), 7.93 (d, J = 5.0 Hz, 2H), 7.77 (d, J = 8.2 Hz, 1H), 7.73 (d, J = 15.6 Hz, 1H), 7.61 (s, 1H), 7.53–7.52 (m, 1H), 7.49 (d, J = 8.0 Hz, 1H), 7.25 (ddd, J = 8.2, 2.7, 0.8 Hz, 1H), 3.85 (s, 3H). ^13^C-NMR (151 MHz, DMSO-*d*_6_) δ 188.9, 159.6, 142.6, 138.9, 135.1, 133.6, 130.9, 130.7, 130.0, 129.0, 128.3, 122.8, 121.1, 119.3, 113.1, 55.4. ESI-MS [M + H]: Calculated for C_16_H_13_ClO_2_: 272.0; Observed: 273.0. HRMS *m*/*z* calculated 273.0682 [M + H]^+^, found 273.0685 [M + H]^+^.

#### 3.1.3. General Method for Pyrazolines Synthesis. Compounds **29**, **30**, **31**

To a solution of the appropriate chalcone (1 equiv.) in 25 mL of absolute ethanol, hydrazine monohydrate (2 equiv.) was added dropwise under stirring. The reaction mixture was heated at reflux for 6–8 h. The hot reaction mixture was then poured into a conical flask containing crushed ice. The crude white precipitate obtained was collected by filtration, washed with water, and dried. Products were recrystallised from ethanol. Compounds **30** and **31** were synthesised according to our previous methods, and characterisation data corresponded to published analytical chemistry information [28]. Please refer to Supplementary Materials.

2-(1′,3′-diphenyl-3,4-dihydro-1′*H*,2*H*-[3,4′-bipyrazol]-5-yl)phenol (**29**). A yellow solid. Yield 64%. ^1^H-NMR (600 MHz, CDCl_3_) δ 8.02 (s, 1H), 7.74–7.70 (m, 4H), 7.50–7.40 (m, 5H), 7.30–7.27 (m, 2H), 7.19 (dd, J = 7.7, 1.5 Hz, 1H), 7.02 (dd, J = 8.2, 1.0 Hz, 1H), 6.90–6.88 (m, 1H), 5.13 (t, J = 9.3 Hz, 1H), 3.58 (dd, J = 16.2, 10.3 Hz, 1H), 3.18 (dd, J = 16.2, 8.8 Hz, 1H). ^13^C-NMR (151 MHz, CDCl_3_) δ 163.6, 154.1, 139.4, 136.2, 136.1, 132.2, 129.6, 129.4, 128.9, 128.8, 127.4, 127.1, 120.0, 119.4, 119.4, 118.7, 118.6, 118.2, 49.3, 41.9. ESI-MS [M + H]: Calculated for C_24_H_20_N_4_O: 380.1; Observed: 381.1. HRMS *m*/*z* calculated 381.1715 [M + H]^+^, found 381.1709 [M + H]^+^.

2-(5-(4-chlorophenyl)-4,5-dihydro-1*H*-pyrazol-3-yl)phenol (**30**). White crystalline solid. Yield 75%. ^1^H-NMR (600 MHz, DMSO-*d*_6_) δ 11.10 (s, 1H), 7.86 (s, 1H), 6.88–7.70 (m, 8H), 4.85 (dd, J = 22.6 Hz, J = 6.4 Hz), 3.63 (dd, J = 16.8 Hz, J = 5.4 Hz), 2.98 (dd, J = 16.6 Hz, J = 5.4 Hz). ^13^C-NMR (151 MHz, MeOD-*d*_4_) δ (ppm) 158.7, 155.6, 142.3, 134.3, 131.2, 129.7, 129.4, 128.9, 120.2, 117.8, 117.0, 63.3, 42.4; ESI-MS [M + H]: Calculated for C_15_H_13_ClN_2_O: 272.1: Observed: 273.1. HRMS *m*/*z* calculated 273.0795 [M + H]^+^, found 273.0795 [M + H]^+^.

2-(5-(4-bromophenyl)-4,5-dihydro-1*H*-pyrazol-3-yl)phenol (**31**). An off-white solid. Yield 68%. ^1^H-NMR (600 MHz, CDCl_3_) δ 7.50–7.48 (m, 2H), 7.28–7.25 (m, 3H, overlapping with solvent residue signal), 7.15 (dd, J = 7.7, 1.5 Hz, 1H), 7.01 (dd, J = 8.2, 1.0 Hz, 1H), 6.88 (td, J = 7.5, 1.1 Hz, 1H), 4.88–4.85 (m, 1H), 3.58 (dd, J = 16.5, 10.7 Hz, 1H), 3.09 (dd, J = 16.5, 9.1 Hz, 1H). ^13^C-NMR (151 MHz, CDCl_3_) δ 156.8, 152.8, 140.6, 131.1, 130.6, 130.4, 129.5, 127.7, 126.8, 120.7, 118.4, 115.8, 115.6, 61.3, 40.9. ESI-MS [M + H]: Calculated for C_15_H_13_BrN_2_O: 316.0; Observed: 317.0. HRMS *m*/*z* calculated 317.0290 [M + H]^+^, found 317.0292 [M + H]^+^.

### 3.2. Antitubercular Screening

#### 3.2.1. MIC under Aerobic Conditions

The antimicrobial activity of compounds against *Mycobacterium tuberculosis* H37Rv grown under aerobic conditions is assessed by determining the minimum inhibitory concentration (MIC) of compound i.e., the concentration required to prevent growth. The assay is based on measurement of growth in liquid medium of a fluorescent reporter strain of H37Rv where the readout is either optical density (OD) or fluorescence. The use of two readouts minimizes problems caused by compound precipitation or autofluoresence. A linear relationship between OD and fluorescence readout has been established justifying the use of fluorescence as a measure of bacterial growth. MICs generated from the OD are reported in summary data. The strain has been fully characterized and is equivalent to the parental strain in microbiological phenotypes and virulence.

#### 3.2.2. Protocol

The MIC of compound was determined by measuring bacterial growth after 5 d in the presence of test compounds. Compounds were prepared as 10-point two-fold serial dilutions in DMSO and diluted into 7H9-Tw-OADC medium (recipe: 4.7 g/L 7H9 base broth, 0.05% *w*/*v* Tween 80,10% *v*/*v* OADC supplement) in 96-well plates with a final DMSO concentration of 2%. Each plate included assay controls for background (medium/DMSO only, no bacterial cells), zero growth (100 µM rifampicin), and maximum growth (DMSO only), as well as a rifampicin dose response curve. Plates were inoculated with *M. tuberculosis* and incubated for 5 days: growth was measured by OD_590_ and fluorescence (Ex 560/Em 590) using a BioTek™ Synergy H4 plate reader. Growth was calculated separately for OD_590_ and RFU. A dose-response curve was plotted as % growth and fitted using the Levenberg–Marquardt algorithm. Concentrations that resulted in 50% and 90% inhibition of growth were determined (IC_50_ and IC_90_, respectively). Raw data is provided and can be used to plot either type of curve.

### 3.3. Antiproliferative Evaluation In Vitro

The cytotoxicity of compounds towards eukaryotic cells was determined using human liver cells (HepG2), non-tumorigenic human mammary epithelial cells (MCF-10A), and breast cancer cells (MDA-MB-468).

#### 3.3.1. HepG2 Cytotoxicity Screening

HepG2 cells were incubated with compounds for 72 h in medium containing either glucose or galactose, and cell viability was measured. The IC_50_ was determined as the concentration of compound causing a 50% decrease in viable cells after 72 h. Compounds were prepared as 10-point three-fold serial dilutions in DMSO. The highest concentration of compound tested was 100 µM where compounds were soluble in DMSO at 10 mM. For compounds with limited solubility, the highest concentration was 50× less than the stock concentration, e.g., 100 µM for 5 mM DMSO stock, 20 µM for 1 mM DMSO stock. HepG2 cells were cultured in complete DMEM (recipe: DMEM medium, 1X penicillin-streptomycin solution, 2 mM Corning Glutogro supplement, 1 mM sodium pyruvate, 10% *v*/*v* Fetal Bovine Serum), inoculated into 384-well assay plates and incubated for 24 h at 37 °C, 5% CO_2_. Compounds were added and cells were cultured for a further 72 h. The final DMSO concentration was 1%. Cell viability was determined using the CellTiter-Glo^®^ Luminescent Cell Viability Assay (Promega, Madison, WI, USA) and measuring relative luminescent units (RLU). The dose-response curve was fitted using the Levenberg–Marquardt algorithm. The IC_50_ was defined as the compound concentration that produced a 50% decrease in viable cells. Each run included staurosporine as a control.

#### 3.3.2. MCF-10A and MDA-468 MTT Cytotoxicity Screening

Cells were purchased from the American Type Culture Collection (Manassas, VA, USA). MDA-468 cells were grown in RPMI-1640 medium with 10% (*v*/*v*) heat-inactivated foetal calf serum and L-glutamine (2 mM) without phenol red. MCF-10A cells were grown in Dulbecco’s modified Eagle’s medium/ Ham’s F-12 medium (1:1) with 5% (*v*/*v*) heat-inactivated foetal calf serum, epidermal growth factor (20 ng/mL), insulin (10 µg/mL), and hydrocortisone (500 ng/mL). Cells were maintained at 37 °C, 5% CO_2_ / 95% air with 100% humidity and passaged every 2–3 days using trypsin EDTA solution (0.25% *w*/*v*). Adhered cells at sub confluence were harvested for experimental use. The medium was aspirated and discarded, and trypsin-EDTA solution (1% *w*/*v*, 1 mL) was added to the cells. After 30 s this was aspirated and immediately replaced by a further 1 mL of trypsin-EDTA solution. The cells were incubated at 37 °C for approximately 5 min or until the cells were visibly non-adherent. The resultant cell suspension was placed in a 25 mL sterile universal container with 10 mL of fresh medium followed by centrifugation (3 min, 3000 rpm, and 4 °C). Old medium was aspirated and replaced with 10 mL of fresh medium. To determine the density of cells in suspension, an aliquot (100 µL) was added to a trypan-blue solution (0.4% *w*/*v*, 100 µL), and the number of viable cells were determined using a Neubauer haemocytometer. The cell suspension was diluted with relevant medium to give a cell count of 2 × 10^3^ cells per mL. Aliquots (100 µL) were dispensed into sterile 96-well microtitre flat-bottomed plates. For the MDA-468 and MCF-10A cell lines the plates were incubated at 37 °C, 5% CO_2_/95% air with 100% humidity for 24 h prior to the addition of test compounds. Test compounds were added within 30 min into the wells from a 100 mM stock solution in DMSO and serially diluted to give final concentrations of 100, 30, 10, 3, 1, 0.1, 0.93, 0.01, 0.003, 0.001, and 0.0003 µM. The final concentration of DMSO did not exceed 0.1% *v*/*v* in each well. The cells were allowed to grow for 96 h at 5%, CO_2_, 37 °C to give 80–90% confluence in the control wells, after which 50 µL of MTT (3-[4,5 dimethylthiazol-2-yl]-2,5-diphenyl tetrazolium bromide, 2 mg mL^−1^) in sterile phosphate buffer was added to each well and the plates were further incubated for 2 h. All medium was aspirated, and the formazan precipitate generated by viable cells was solubilised by 150 µL of DMSO. All the plates were vortexed, and the absorbance at 540 nm was determined using a Molecular Devices SpectraMax M5 plate reader. Results were expressed as a percentage of the control value versus the negative logarithm of the molar drug concentration range using Graph Pad Prism. Relative toxicities of each compound within each cell line were expressed as 50% of growth inhibition (IC50). All determinations were carried out in quadruplicate.

### 3.4. Molecular Docking

The molecular docking was conducted using two different platforms AutoDock Vina ver 1.12 [29] with VegaZZ ver 3.2.3.28 as a graphical user interface (GUI) and GOLD [30] with Hermes ver 2024.1.0 as GUI. [34] Protein target structures for docking were downloaded from the Protein Databank (PDB) website as individual entries, namely, PtpA (PDB ID 1U2Q) and PtpB (PDB ID 2OZ5). Protein files are initially processed using PDBfixer script to remove water molecules and unnecessary ligands, and to add missing residues. The three-dimensional structures of all small molecules were generated by converting ChemDraw structures using Chem3D ver 22.0.0 software and were further processed using VegaZZ. The protonation state of all protein and ligand ionizable groups were set to reflect the pH 7 environment using VegaZZ. The binding sites docking were set using of the bound ligands 7XY and GOL determined either manually for AutoDock Vina or using Ligsite cavity detection algorithm for GOLD (Table 3).

The exhaustiveness AutoDock Vina parameter was set to 50, and five favourable docking poses were set in the VegaZZ Virtual Screening Script. The GOLD parameters were set to default values, except the GoldScore was chosen as the scoring method, and the genetic algorithm’s (GA) search efficiency value was set to 200% to deliver high predictive accuracy with a speed trade-off.

The docking was validated by redocking the ligands from the crystal structure back into the binding site. Although only one RMSD appears to be acceptable as it is below 2 Å (Table 3), the docking deemed to be correct as the ligands bind in the right places of the binding site and in a correct orientation. The reproducibility of the crystal structures cannot be achieved in these two structures due to different reasons. The ligand in the binding site of the PtpA structure is glycerol, and as a small molecule, it is not predicted to form enough specific interactions that would ensure the reproducibility of the ligand conformation. In case of PtpB, there are two copies of the inhibitor in the binding site of the crystal structure, while the docking can be carried out with one molecule at the time. Therefore, the interligand interactions from the crystal structures are missing to ensure that the docking pose closely resembles the inhibitor conformation in the crystal structure.

**Table 3:** Coordinates of the centres and sizes of the box and cavities used in the docking process by AutoDock Vina and GOLD, respectively. RMSD values indicate the discrepancy between conformations of the inhibitor in the crystal structure and the most favourable docking pose.

PDB ID	AutoDock Vina			GOLD		
	Box Centre (Å)	Cube Size (Å)	RMSD (Å)	Cavity Centre (Å)	Cavity Radius (Å)	RMSD (Å)
1U2Q	14.0; −7.0; 3.5	24	4.0	14.6; −6.4; 3.7	8.6	3.65
2OZ	7.0; 64.0; 4.0	24	1.76	6.0; 60.7; 6.2	15.4	2.34

## 4. Conclusions

Chalcones represent an easily accessible synthetic framework endowed with significant biological, and most importantly, anti-tubercular properties. The pyridine-based chalcones reported here had growth-inhibition activities in the low micromolar region against H37Rv strains with dichloro-phenyl- (**14**), pyrene-1-yl (**20**)-, and biphenyl-4-yl (**21**)-including compounds exhibiting IC_90_ values ranging from 8.9–29 µM. The obvious downside associated with Scaffold A pyridyl-chalcones being used as anti-infective lead-compounds might be their cytotoxicity, which was observed in the cell lines used in this study. However, this might be an advantage if selected compounds were solely to be deployed as antiproliferative agents. Indeed, trimethoxy-phenyl (**16**) and anthracene-9-yl (**19**) pyridyl-chalcones exhibited IC_50_ values of 0.7 and 0.3 µM, respectively, against MDA468 cells. A marginally improved toxicity profile can be seen for Scaffold B chalcones **25** and **26**, which were active against *M. tuberculosis* H37Rv at concentrations as low as 28 µM and were ~five-fold less toxic to HepG2 cells compared to Scaffold A derivatives. This study indicates that the presence of an electron withdrawing group (i.e., halo-phenyl group or pyridine unit) in ring B is required for the anti-tubercular activity of the chalcones. As chalcones might serve as a scaffold to construct higher chemical structures, we employed **27** (IC_50 H37Rv_ = >100 µM) to synthesise the tri-cyclic pyrazoline **29**, which was 10-fold more active (IC_50 H37Rv_ = 11 µM) than its parent compound (**27**), and circa two-fold more active than mycobactin analogue **31**. Molecular modelling experiments using pyridyl-chalcones and *M. tuberculosis* Mtb PtpA (PDB ID: 1U2P) and Mbt PtpB (PDB ID: 2OZ5) as targets revealed that compound **20** had a very favourable docking score within the PtpB site.

## Figures and Tables

**Table 1:** Anti-tubercular and cytotoxic activity of the chalcone analogues. Values are expressed in µM units.

			Cytotoxicity		
	Anti-Tubercular Activity *^a^*		HepG2 *^b^*	MCF10A *^c^* Normal Breast Cells	MDA468 *^c^* Breast Cancer Cells
	*M. tuberculosis* (H37Rv)				
CMP ID	IC_90_	IC_50_ (SD)	IC_50_	IC_50_	IC_50_
**14**	28	12 (3.5)	2.5	ND	ND
**15**	77	24 (1.4)	11	ND	ND
**16**	59	19 (1.4)	2.0	1.5	0.7
**17**	>100	>100	>100	>100	30
**18**	>100	>100	22	16	8
**19**	48	10 (1.4)	2.7	4.4	0.3
**20** *^d^*	22	8.8 (0.3)	1.8	2	4
**21** *^d^*	8.9	3.9 (1.1)	4.5	7	3.5
**22**	59	44.5 (1.2)	13	ND	ND
**23**	>100	-	25	ND	ND
**24**	97	>100	14	ND	ND
**25**	29	22 (7.0)	14	ND	ND
**26**	28	20 (5.6)	13	ND	ND
**27**	>100	>100	74	ND	ND
**28**	65	ND	ND	5.2	0.7
**2** *^d^* (DMU-135)	>100	ND	ND	2.3	0.006 [12]
**29**	>25	11 (1.8)	46.5	ND	ND
**30**	73	23 (2.1)	19.5	ND	ND
**31**	34	23 (1.4)	24.5	ND	ND
**Combretastatin A-4**	ND	ND	ND	0.0016	0.003
**Rifampicin**	0.0087	0.0044	ND	ND	ND

*^a^* Inhibitory concentrations (ICs) were determined after a 5-day incubation period. IC_90_ and IC_50_ are the compound concentrations that would inhibit the growth of 90% and 50%, respectively, of the tested bacterial isolates. SD = Standard Deviation. ND = Not determined. *^b^* The IC_50_ is determined as the concentration of compound causing a 50% decrease in viable cells after 72 h. *^c^* Relative toxicities of each compound within each cell line were expressed as 50% of growth inhibition (IC_50_). All determinations were carried out in quadruplicate. *^d^* Analogues previously investigated for their CYP1 inhibitory properties that were tested here, for the first time, as anti-tubercular agents. DMU-135 anti-proliferative activity against MDA-MB-468 was previously reported [12,25].

**Table 2:** Docking scores of PtpB inhibitor **13**, pyridyl chalcones **19**, **20**, and **21**, and CYP1 inhibitor **2** (DMU-135), against *M. tuberculosis* Mtb PtpA (PDB ID: 1U2P) and Mbt PtpB (PDB ID: 2OZ5) as targets. The docking scores of the most favourable poses were obtained by AutoDock Vina using VegaZZ as a graphical user interface, while the GoldScore fitness value were estimated using GOLD software. v. 2024.1.0. The K_d_ values were estimated based on Vina docking scores, assuming room temperature (T = 298.15 K).

	Vina Docking Score (kcal/mol)		K_d_ (µM)		GoldScore Fitness	
Chalcone	PtpA 1U2P	PtpB 2OZ5	PtpA 1U2P	PtpB 2OZ5	PtpA 1U2P	PtpB 2OZ5
**13**	−8.2	−9.2	0.96	0.18	54.95	60.47
**19**	−7.3	−8.9	4.4	029	43.8	55.1
**20**	−8.1	−9.6	1.1	0.09	49.14	54.88
**21**	−6.9	−8	8.6	1.3	44.06	47.12
**2**	−7.3	−7.6	4.4	2.6	44.73	61.63

## Data Availability

Spectral data are available in the Supplementary Material.

## Supplementary Materials

The following supporting information can be downloaded at: https://www.mdpi.com/article/10.3390/molecules29194539/s1, Spectral data for selected compounds.
