# Supplementary material for: Design and Synthesis of Pyridyl and 2-Hydroxyphenyl Chalcones with Antitubercular Activity
Source: Molecules. 2024 Sep 24;29(19):4539. doi: 10.3390/molecules29194539 (PMC11478287; doi:10.3390/molecules29194539)
Supplement: Supplementary file 1 [file molecules-29-04539-s001.zip › molecules-3108598-supplementary.pdf]

Supporting Information

Design and synthesis of pyridyl and 2-hydroxyphenyl chalcones with remarkable antituberculosis activity.

Kelphina Aziafor,[1] Ketan Ruparelia, Brandon Moulds,[1] [1] Mire Zloh,[2,3] Tanya Parish,[4] Federico Brucoli[1]*

[1]Leicester School of Pharmacy, De Montfort University, Leicester, LE1 9BH, UK.

[2]Faculty of Pharmacy, University Business Academy, Novi Sad, 2100, Serbia

[3]UCL School of Pharmacy, UCL, London WC1N 1AX, UK

[4]Center for Global Infectious Disease Research, Seattle Children's Research Institute, 307 Westlake Avenue North, Suite 500, Seattle, USA.

*Corresponding author: Dr F. Brucoli.

E-mail:federico.brucoli@dmu.ac.uk; orcid.org/ 0000-0001-8661-2910

NMR Spectral data for selected compounds

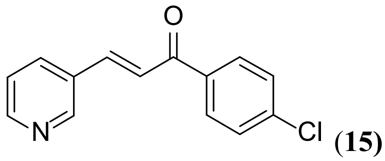

(15)

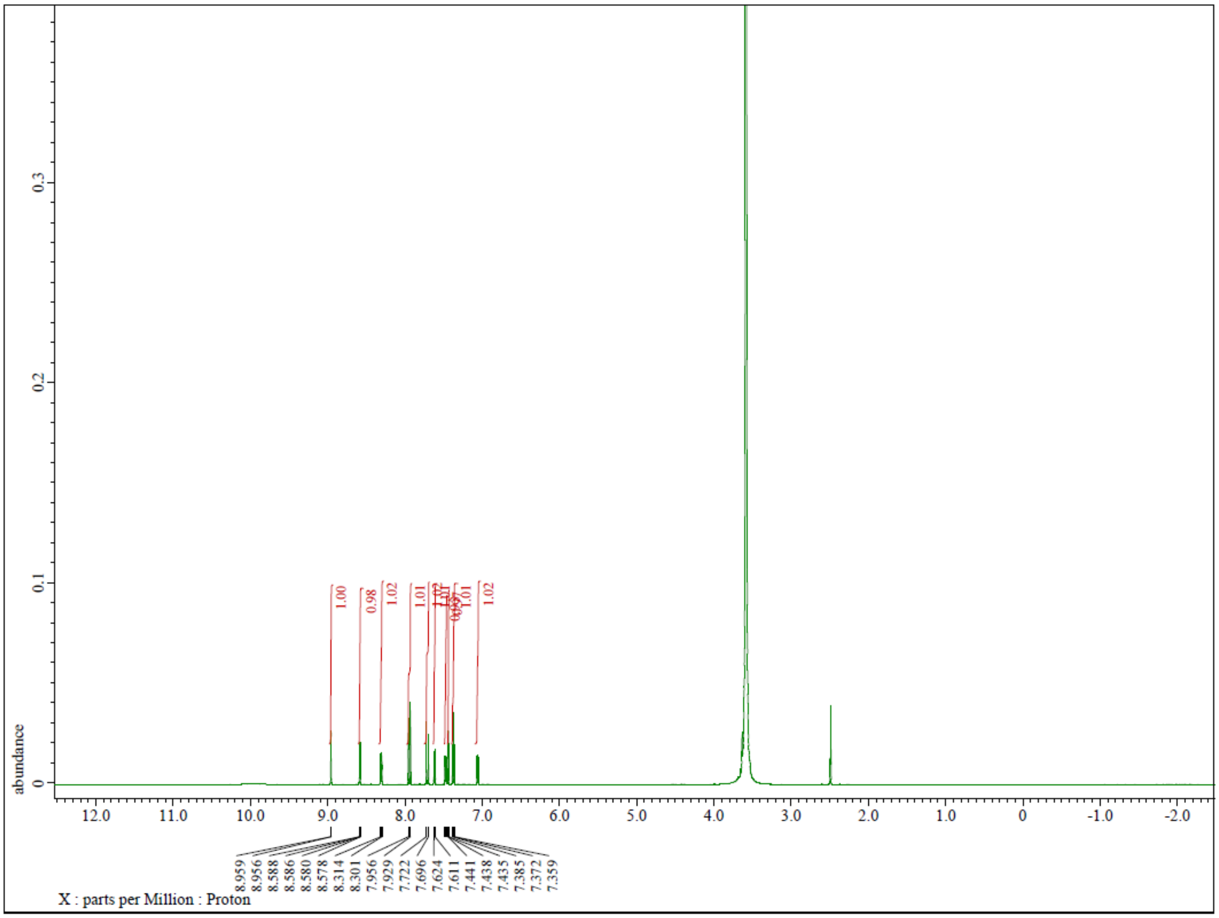

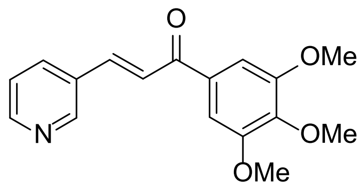

(**16**)

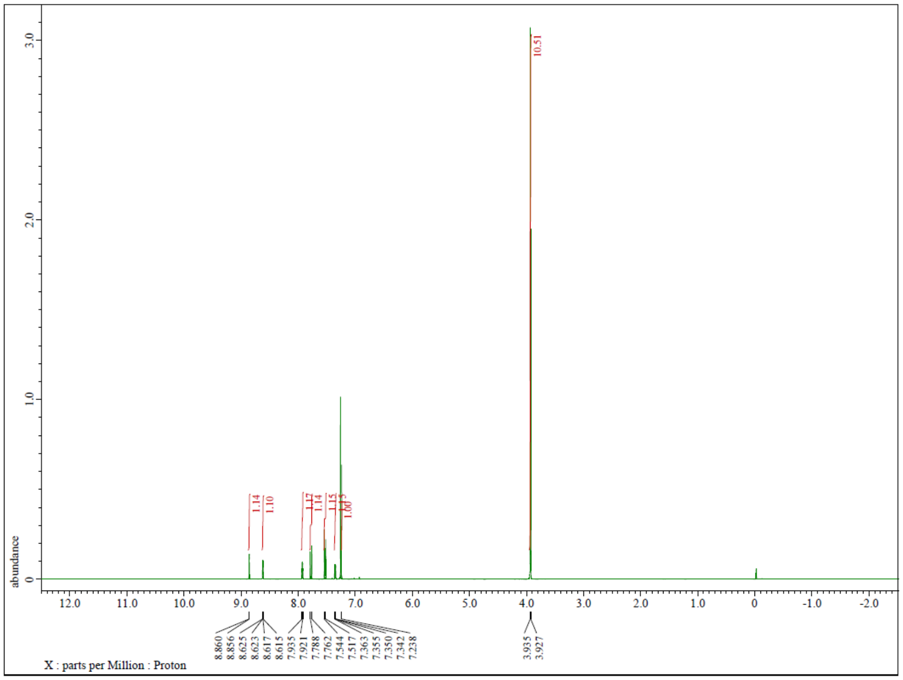

abundance
X : parts per Million : Proton

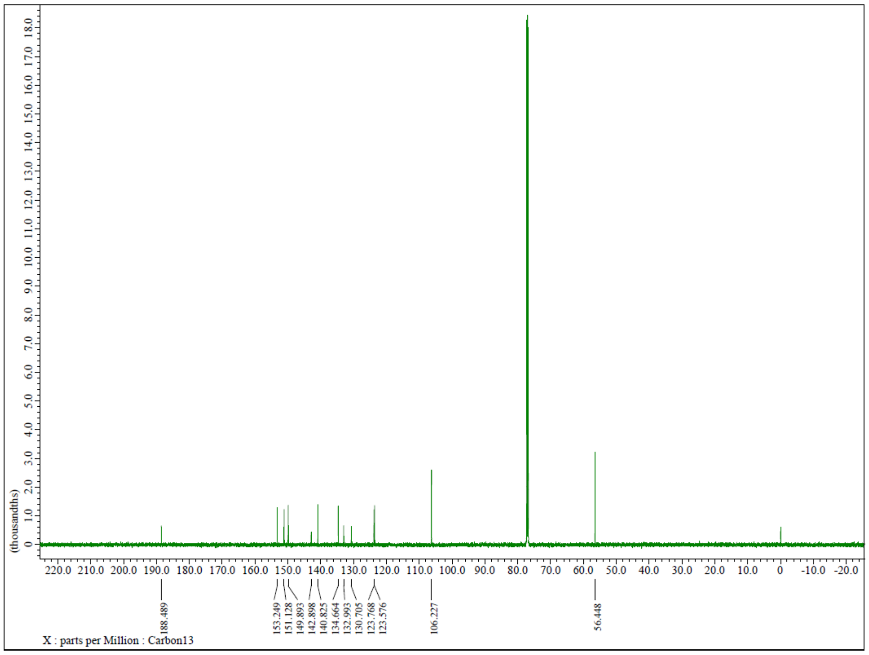

(thousandths)
X : parts per Million : Carbon13

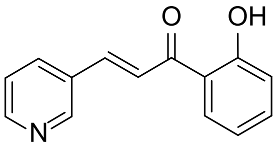

(**18**)

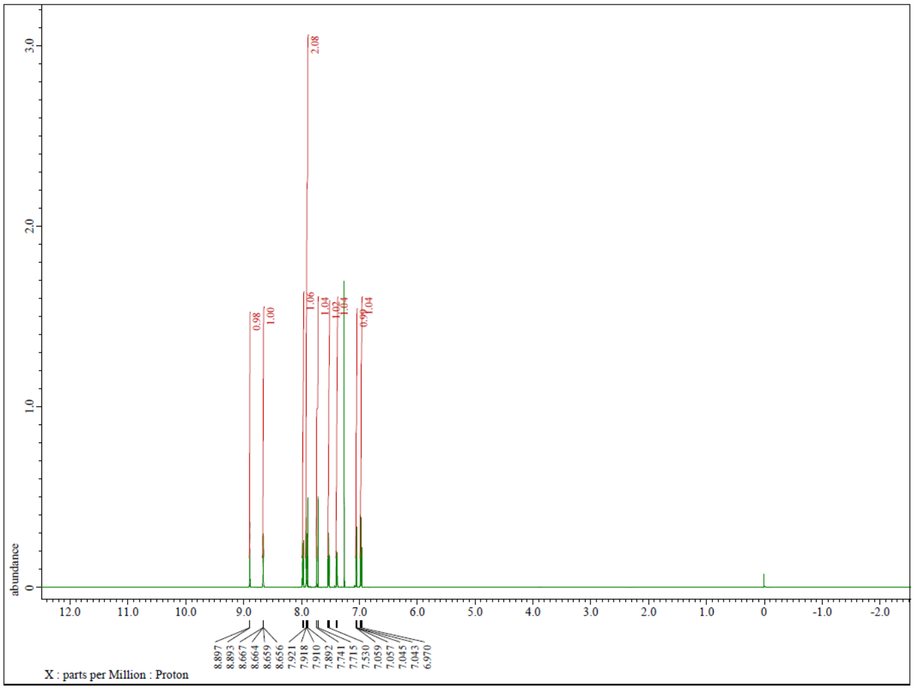

abundance
X : parts per Million : Proton

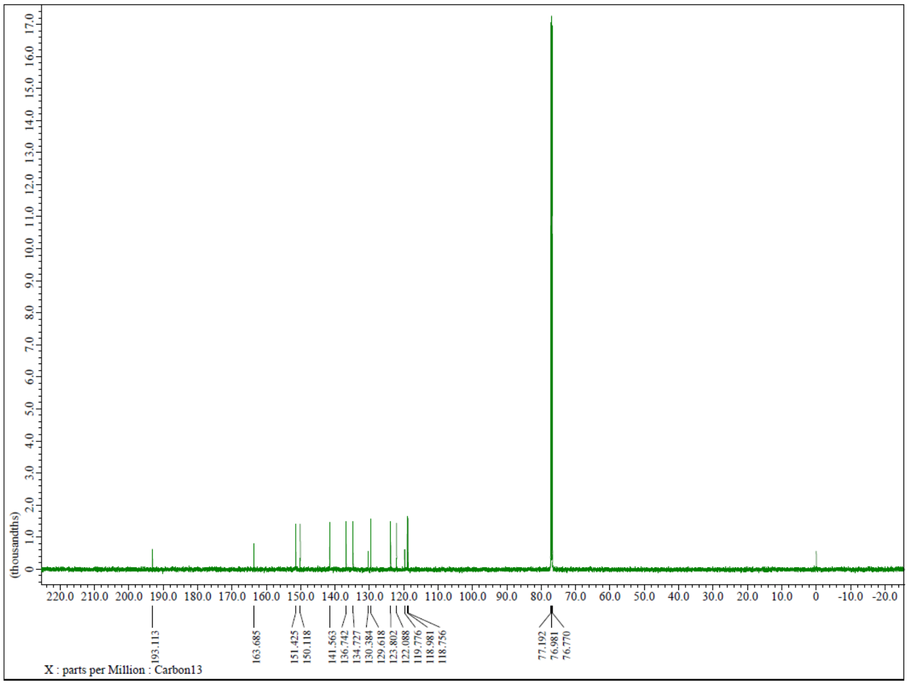

(thousandths)
X : parts per Million : Carbon13

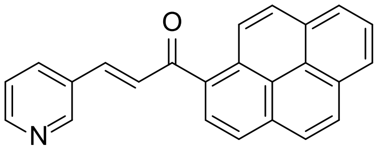
O
N

(**20**)

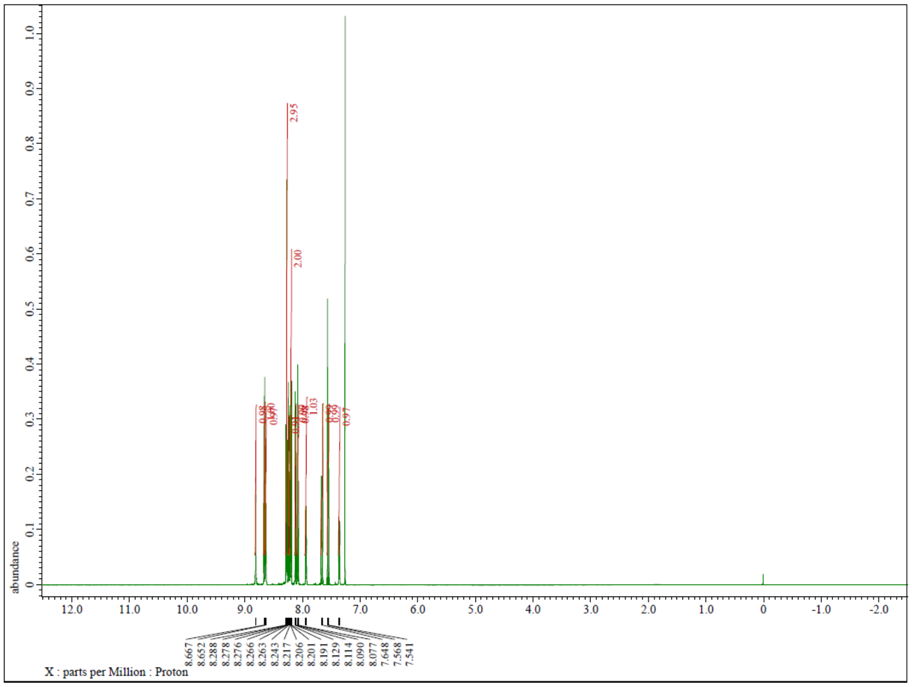
abundance
X : parts per Million : Proton
8.667
8.652
8.288
8.278
8.276
8.266
8.263
8.243
8.217
8.206
8.201
8.191
8.129
8.114
8.090
8.077
7.648
7.568
7.541

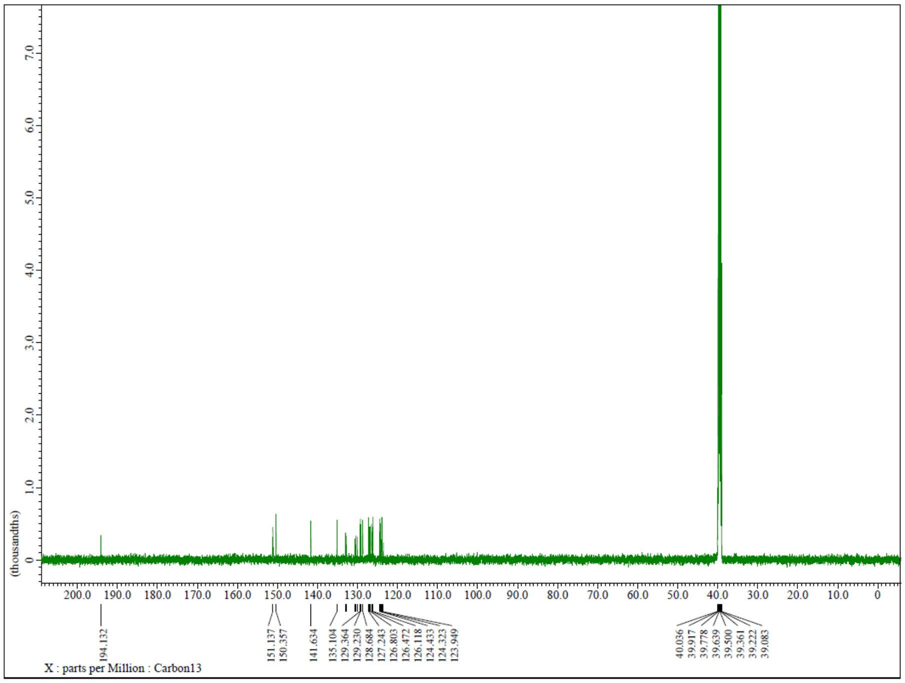
(thousandths)
X : parts per Million : Carbon13
194.132
151.137
150.357
141.634
135.104
129.364
129.230
128.684
127.243
126.803
126.472
126.118
124.433
124.323
123.949
40.036
39.917
39.778
39.639
39.500
39.361
39.222
39.083

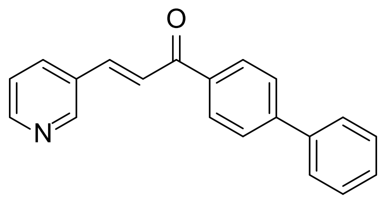

(**21**)

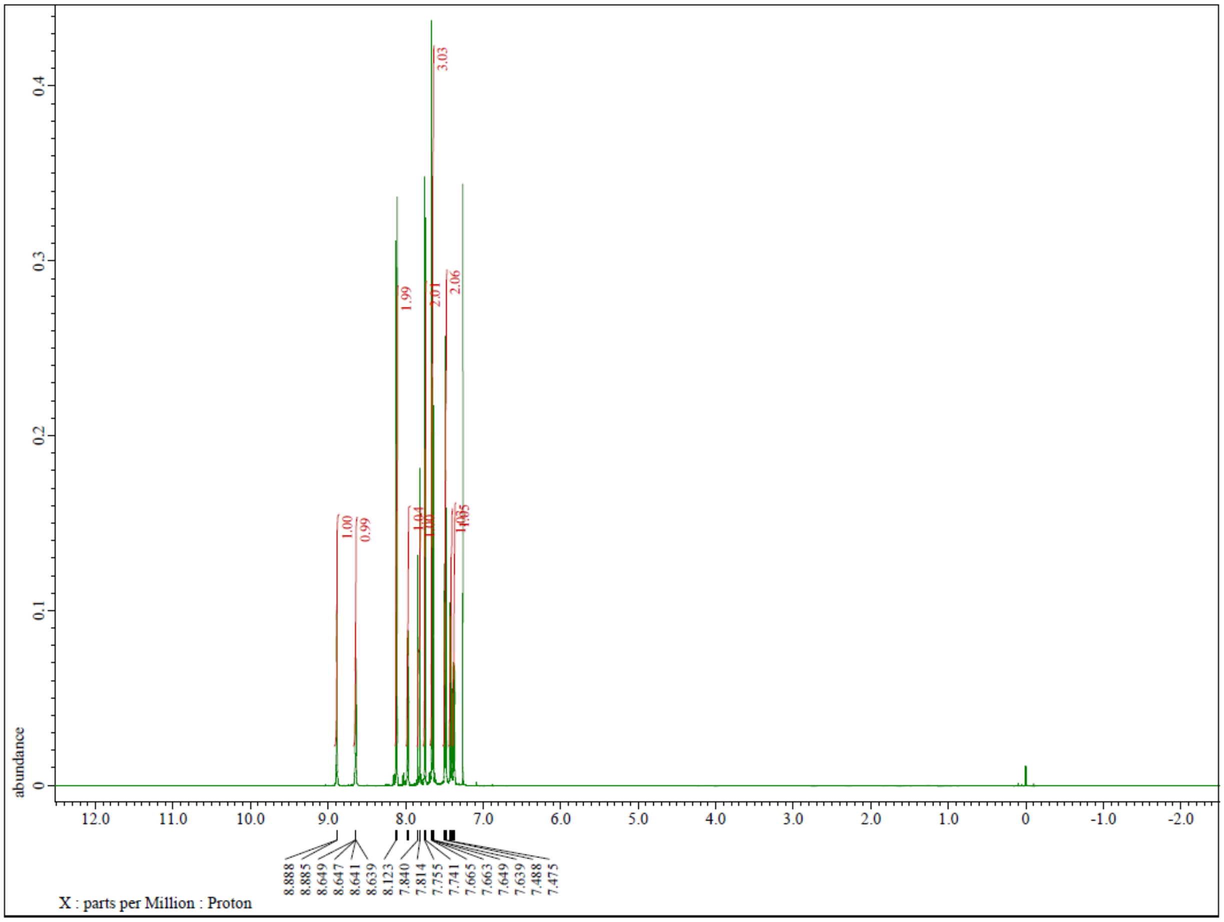

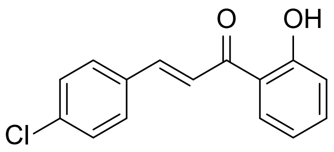
O
OH
Cl
(**23**)

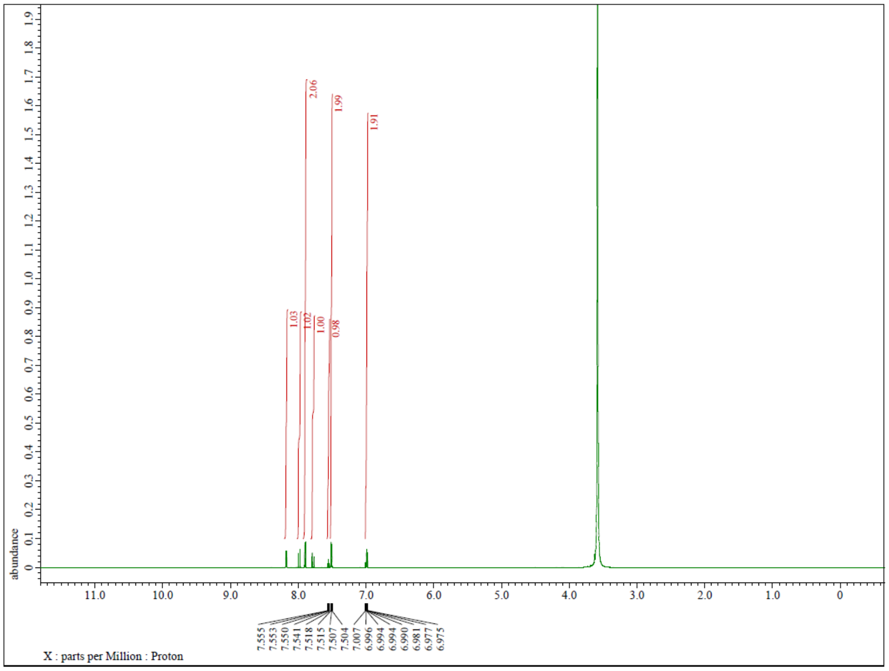
abundance
1.03
1.02
2.06
1.00
0.98
1.99
1.91
7.555
7.553
7.550
7.541
7.518
7.515
7.507
7.504
7.007
6.996
6.994
6.994
6.990
6.981
6.977
6.975
X : parts per Million : Proton

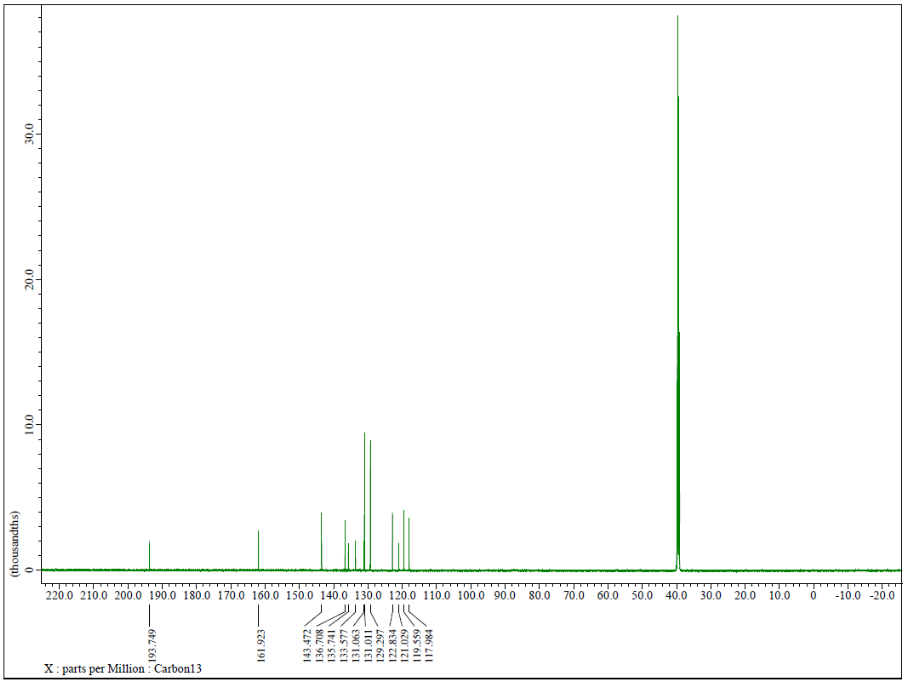
(thousandths)
193.749
161.923
143.472
136.708
135.741
133.577
131.063
131.011
129.297
122.834
121.029
119.559
117.984
X : parts per Million : Carbon13

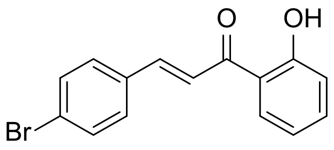
O
OH
Br

(**24**)

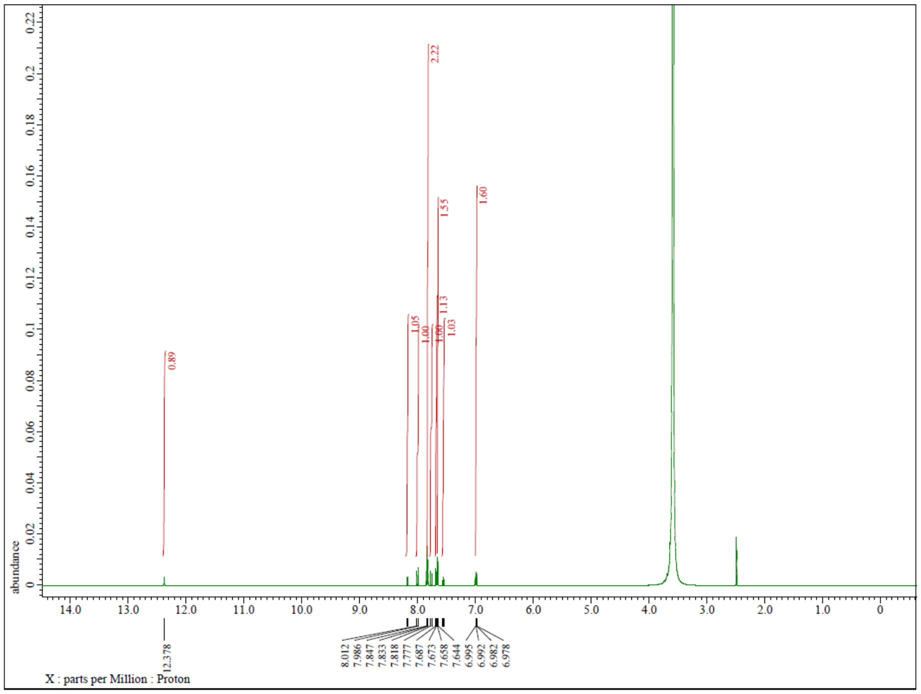
abundance
0.89
1.05
1.00
2.22
1.00
1.55
1.13
1.03
1.60
12.378
8.012
7.986
7.847
7.833
7.818
7.777
7.687
7.673
7.658
7.644
6.995
6.992
6.982
6.978
X : parts per Million : Proton

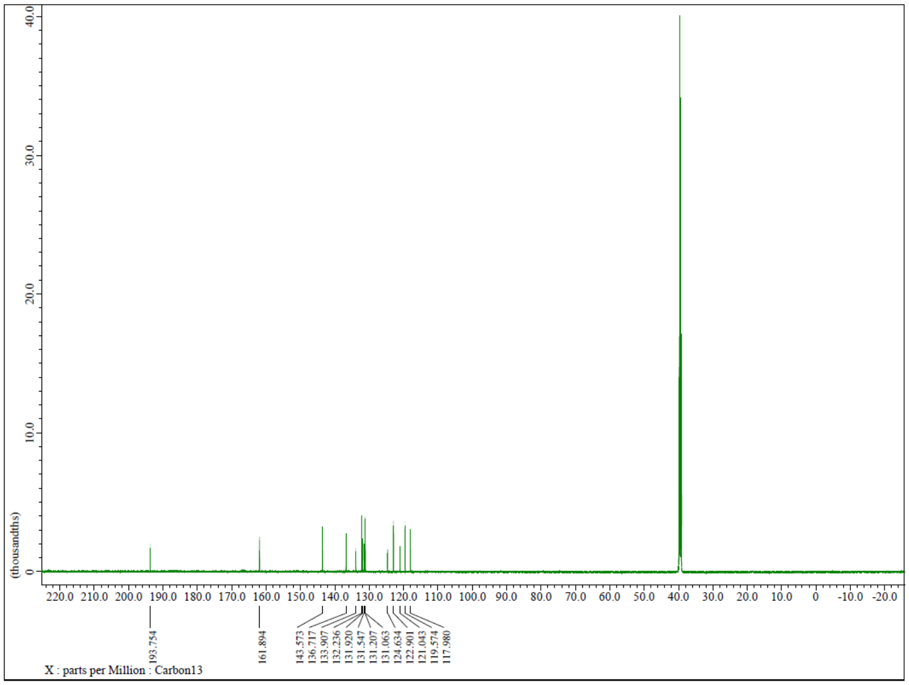
(thousandths)
193.754
161.894
143.573
136.717
133.907
132.236
131.920
131.547
131.207
131.063
124.634
122.901
121.043
119.574
117.980
X : parts per Million : Carbon13

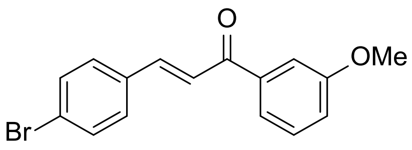
OMe
Br
O

(**25**)

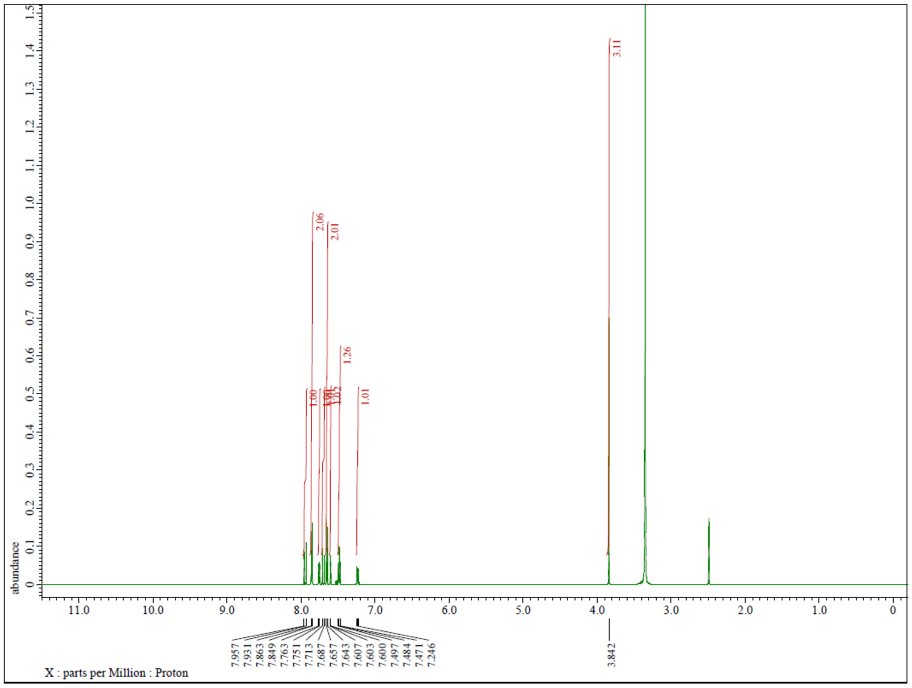
abundance
X : parts per Million : Proton
7.957
7.931
7.863
7.849
7.763
7.751
7.713
7.687
7.657
7.643
7.607
7.603
7.600
7.497
7.484
7.471
7.246
3.842
3.11
2.06
2.01
1.26
1.00
1.01

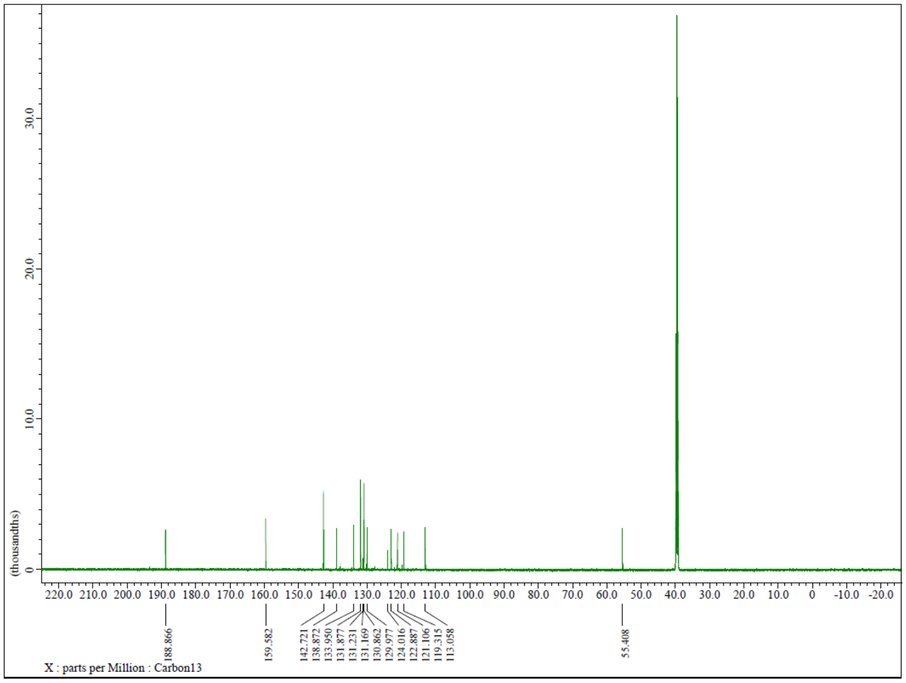
(thousandths)
X : parts per Million : Carbon13
188.866
159.582
142.721
138.872
133.950
131.877
131.231
131.169
130.862
129.977
124.016
122.887
121.106
119.315
113.058
55.408

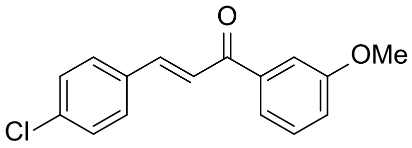
O
OMe
Cl

(**26**)

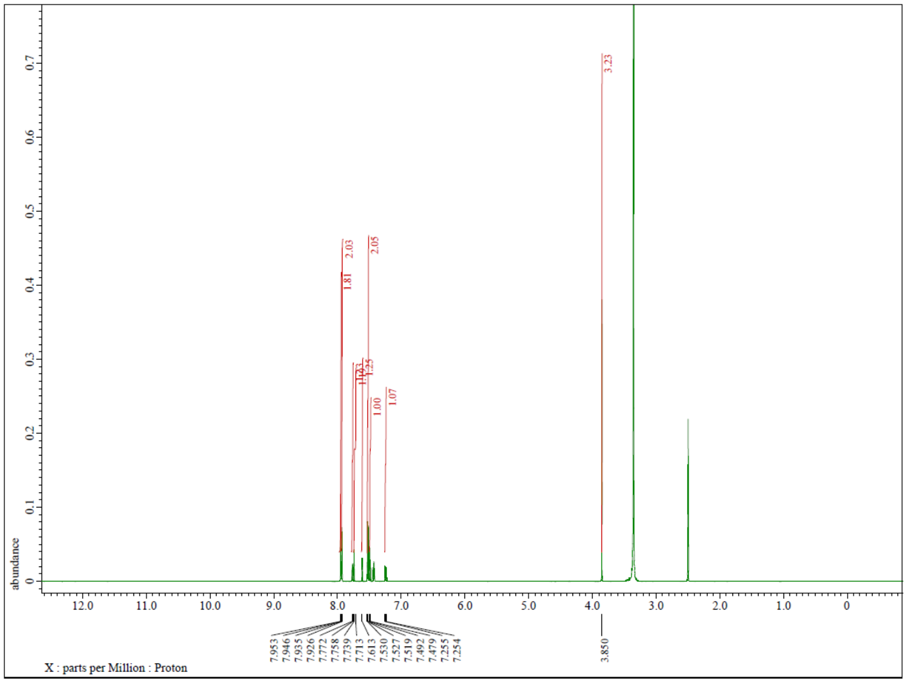
X : parts per Million : Proton
abundance

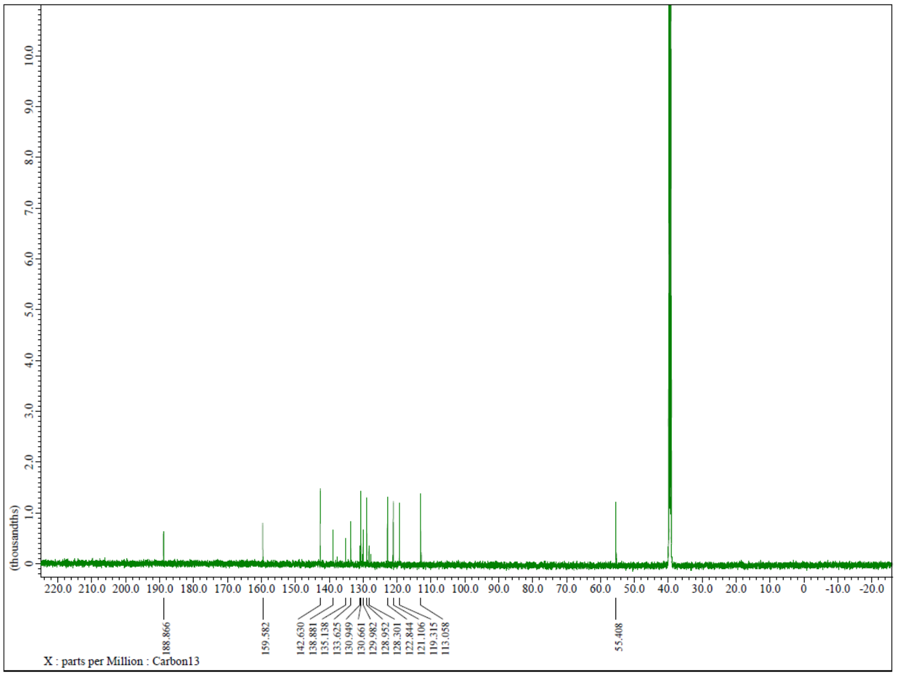
X : parts per Million : Carbon13
(thousandths)

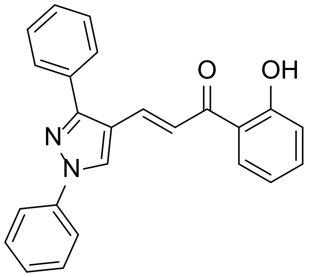
O
OH
N
N

(**27**)

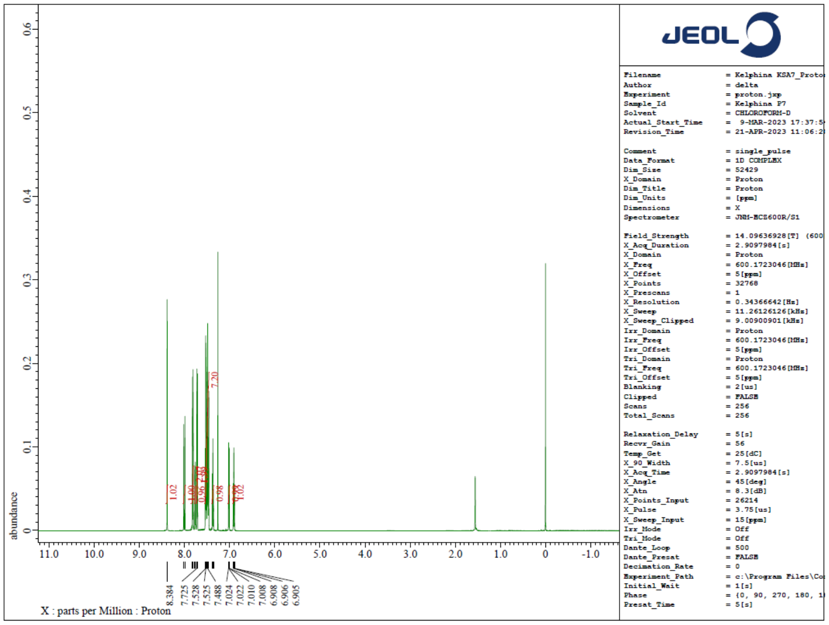
JEOL
abundance
X : parts per Million : Proton
8.384
7.725
7.528
7.525
7.488
7.024
7.022
7.010
7.008
6.908
6.906
6.905

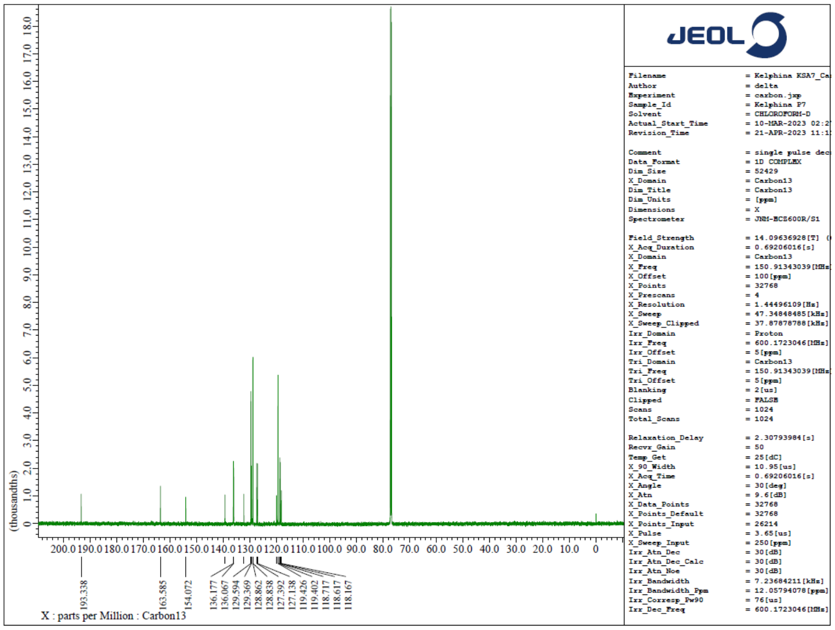
JEOL
(thousandths)
X : parts per Million : Carbon13
193.338
163.585
154.072
136.177
136.067
129.594
129.369
128.862
128.838
127.392
127.138
119.426
119.402
118.717
118.617
118.167

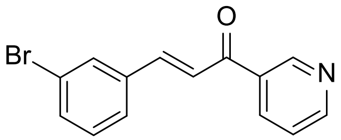

Br
O
N

(28)

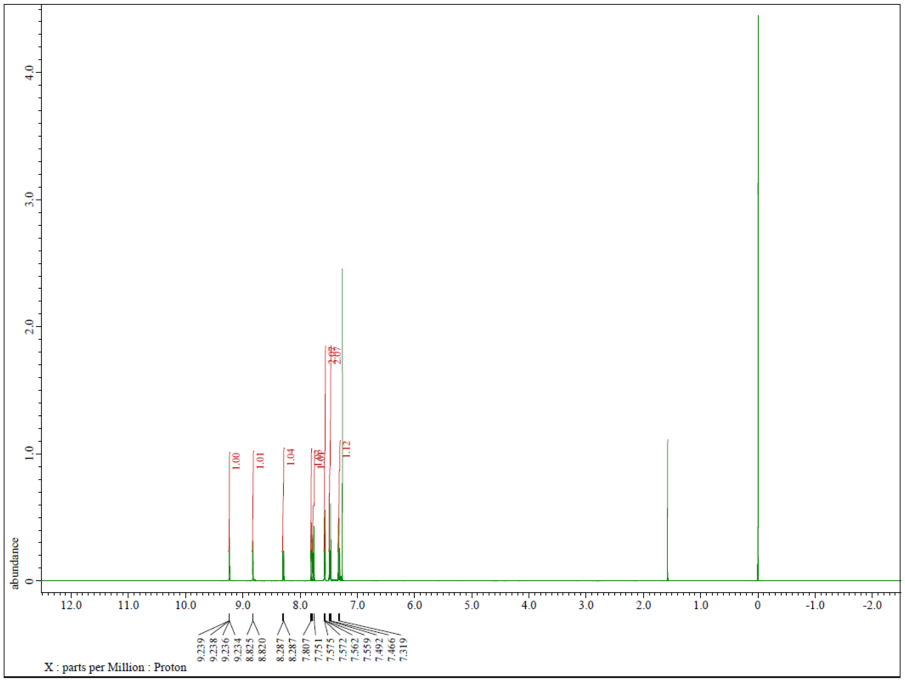

abundance
1.00
1.01
1.04
1.12
9.239
9.238
9.236
9.234
8.825
8.820
8.287
8.287
7.807
7.751
7.575
7.572
7.562
7.559
7.492
7.466
7.319
X : parts per Million : Proton

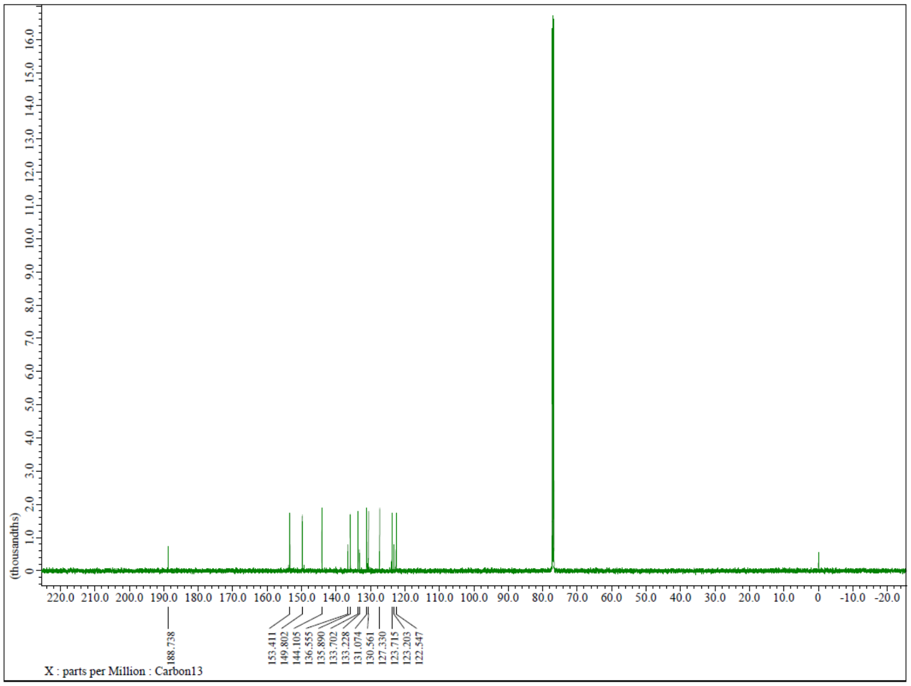

(thousandths)
188.738
153.411
149.802
144.105
136.555
135.890
133.702
133.228
131.074
130.561
127.330
123.715
123.203
122.547
X : parts per Million : Carbon13

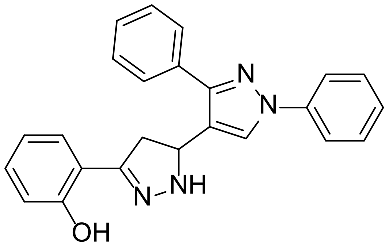
N
N
NH
N
OH

(**29**)

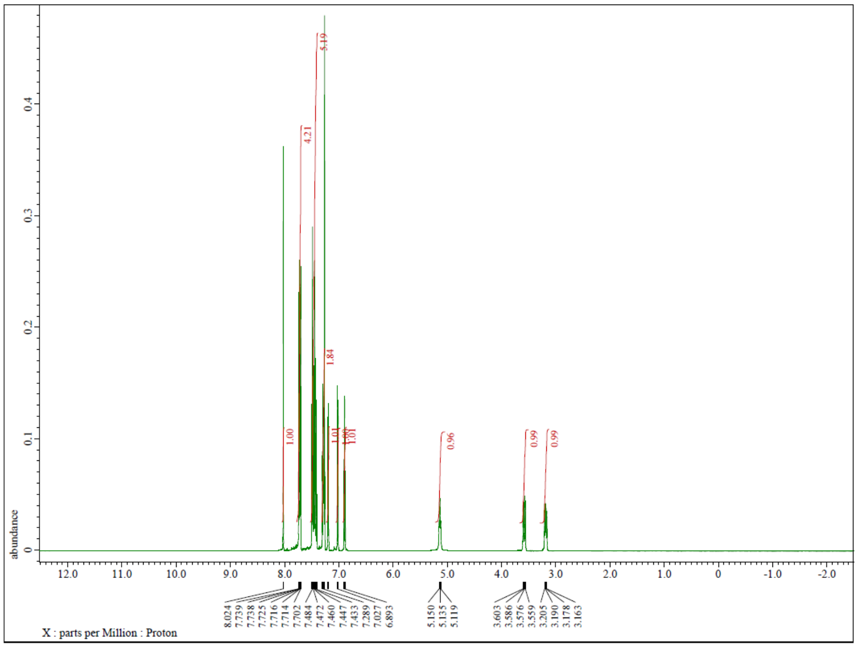
X : parts per Million : Proton
abundance

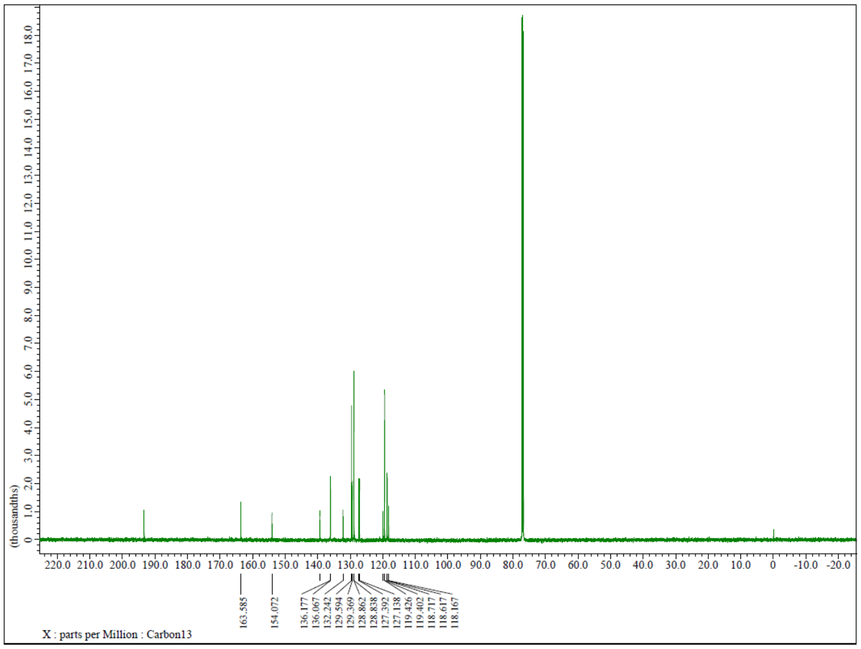
X : parts per Million : Carbon13
(thousandths)

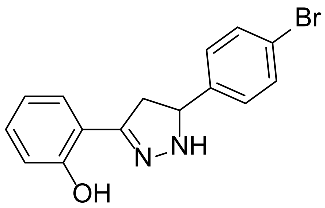
Br
NH
N
OH

(**31**)

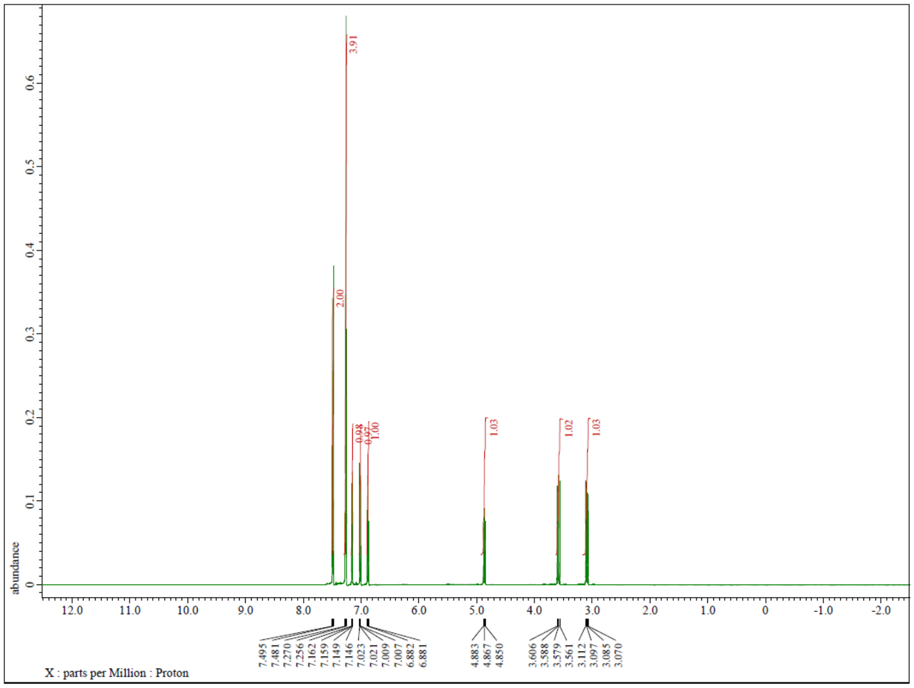
abundance
7.495
7.481
7.270
7.256
7.162
7.159
7.149
7.146
7.023
7.021
7.009
7.007
6.882
6.881
4.883
4.867
4.850
3.606
3.588
3.579
3.561
3.112
3.097
3.085
3.070
X : parts per Million : Proton

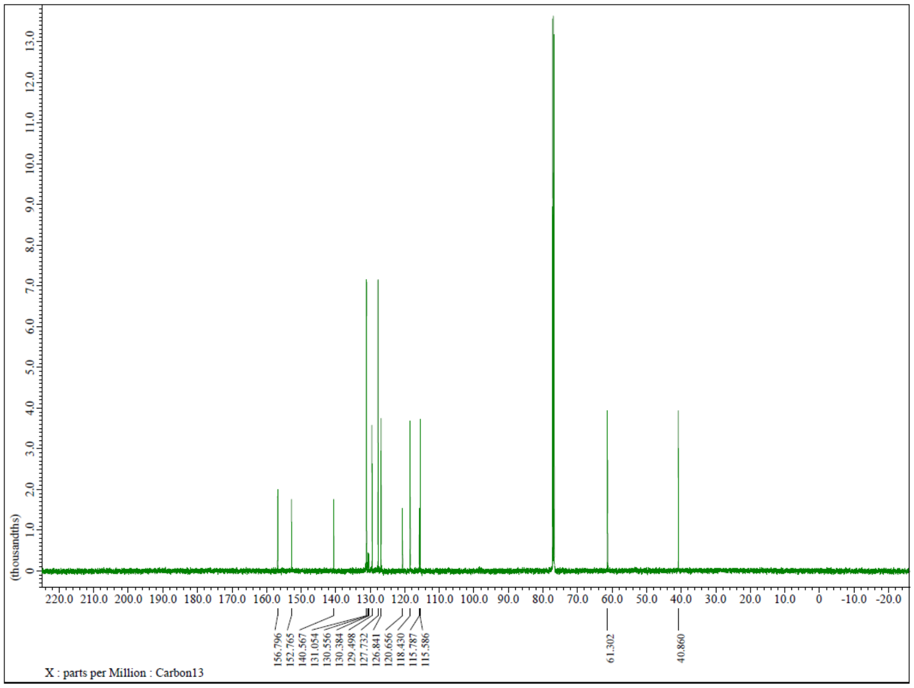
(thousandths)
156.796
152.765
140.567
131.054
130.556
130.384
129.498
127.732
126.841
120.656
118.430
115.787
115.586
61.302
40.860
X : parts per Million : Carbon13

Accurate mass determination of selected compounds

14

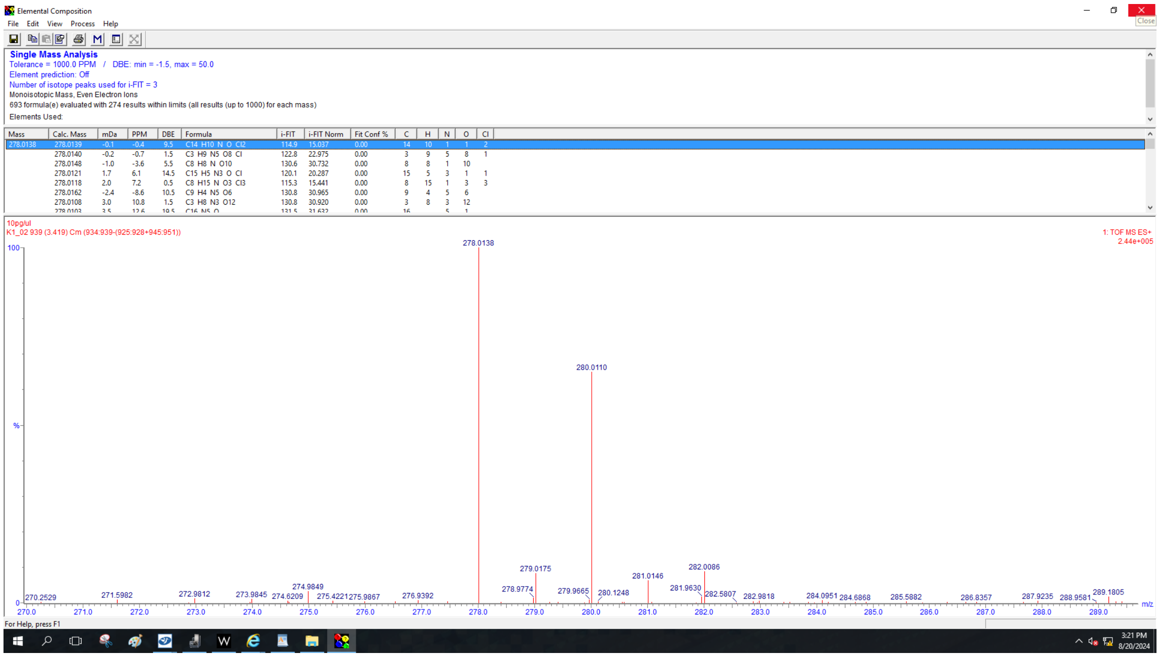

15

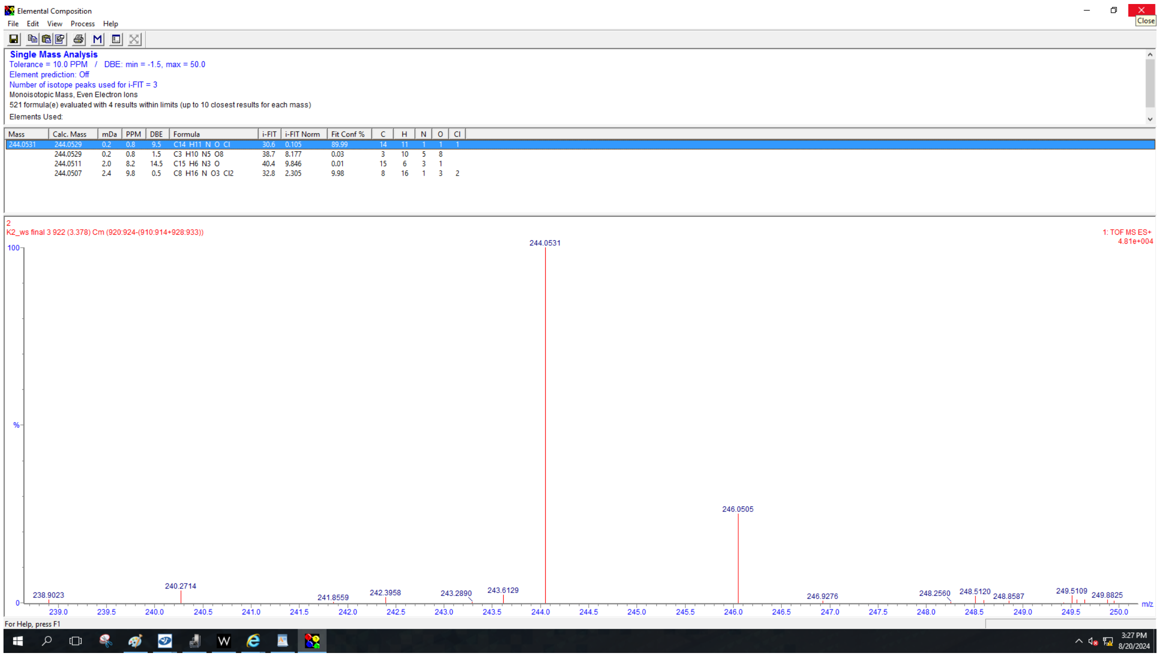

16

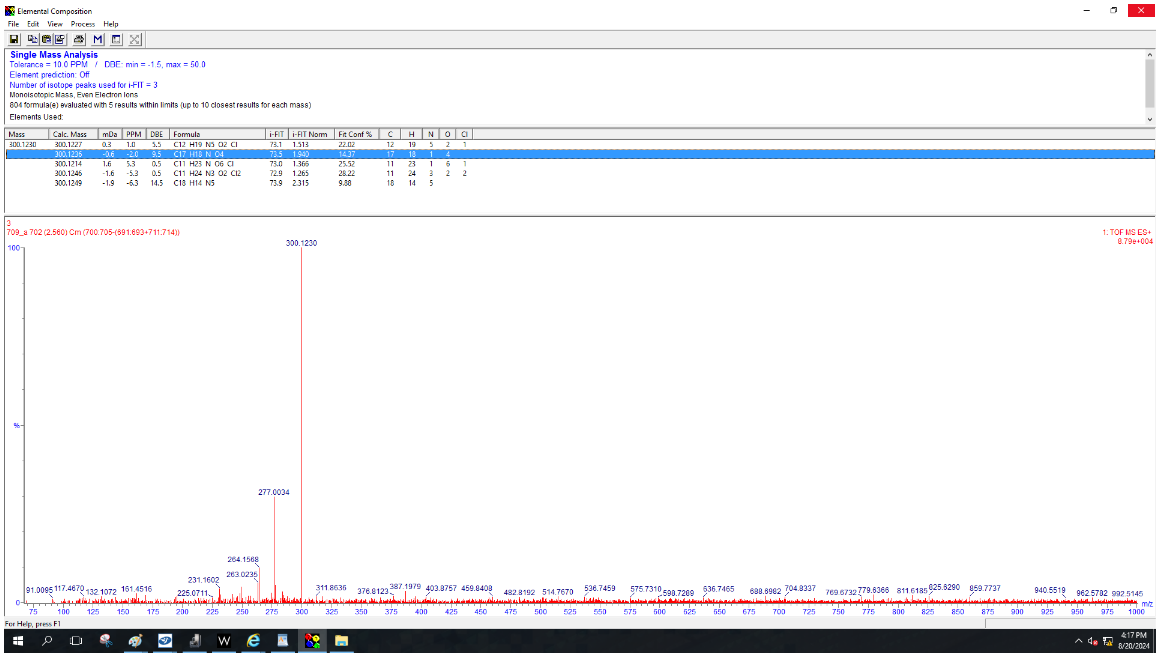
Elemental Composition
File Edit View Process Help
Single Mass Analysis
Tolerance = 10.0 PPM / DBE: min = -1.5, max = 50.0
Element prediction: Off
Number of isotope peaks used for i-FIT = 3
Monoisotopic Mass, Even Electron Ions
804 formula(e) evaluated with 5 results within limits (up to 10 closest results for each mass)
Elements Used:
Mass Calc. Mass mDa PPM DBE Formula i-FIT i-FIT Norm Fit Conf % C H N O Cl
300.1230 300.1227 0.3 1.0 5.5 C12 H19 N5 O2 Cl 73.1 1.513 22.02 12 19 5 2 1
300.1236 -0.6 -2.0 9.5 C17 H18 N O4 73.5 1.940 14.37 17 18 1 4
300.1214 1.6 5.3 0.5 C11 H23 N O6 Cl 73.0 1.366 25.52 11 23 1 6 1
300.1246 -1.6 -5.3 0.5 C11 H24 N3 O2 Cl2 72.9 1.265 28.22 11 24 3 2 2
300.1249 -1.9 -6.3 14.5 C18 H14 N5 73.9 2.315 9.88 18 14 5
3
709_a 702 (2.560) Cm (700:705-(691:693+711:714))
1: TOF MS ES+
8.79e+004
300.1230
277.0034
264.1568
263.0235
231.1602
225.0711
m/z
For Help, press F1

18

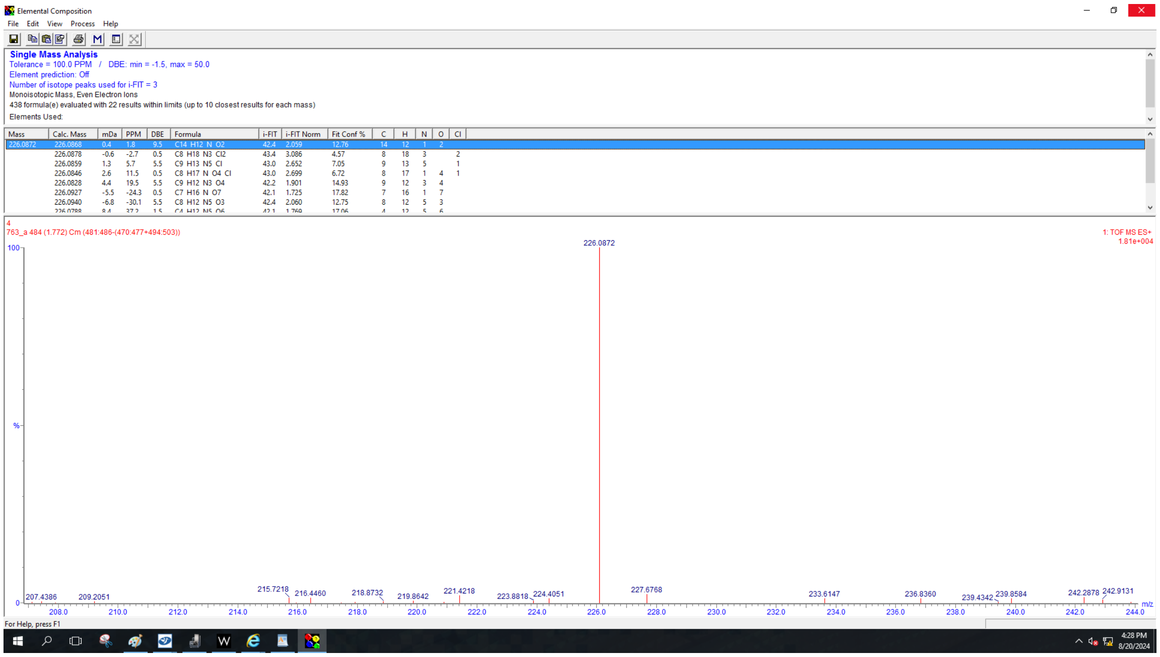
Elemental Composition
File Edit View Process Help
Single Mass Analysis
Tolerance = 100.0 PPM / DBE: min = -1.5, max = 50.0
Element prediction: Off
Number of isotope peaks used for i-FIT = 3
Monoisotopic Mass, Even Electron Ions
438 formula(e) evaluated with 22 results within limits (up to 10 closest results for each mass)
Elements Used:
Mass Calc. Mass mDa PPM DBE Formula i-FIT i-FIT Norm Fit Conf % C H N O Cl
226.0872 226.0868 0.4 1.8 9.5 C14 H12 N O2 42.4 2.059 12.76 14 12 1 2
226.0878 -0.6 -2.7 0.5 C8 H18 N3 Cl2 43.4 3.086 4.57 8 18 3 2
226.0859 1.3 5.7 5.5 C9 H13 N5 Cl 43.0 2.652 7.05 9 13 5 1
226.0846 2.6 11.5 0.5 C8 H17 N O4 Cl 43.0 2.699 6.72 8 17 1 4 1
226.0828 4.4 19.5 5.5 C9 H12 N3 O4 42.2 1.901 14.93 9 12 3 4
226.0927 -5.5 -24.3 0.5 C7 H16 N O7 42.1 1.725 17.82 7 16 1 7
226.0940 -6.8 -30.1 5.5 C8 H12 N5 O3 42.4 2.060 12.75 8 12 5 3
4
763_a 484 (1.772) Cm (481:486-(470:477+494:503))
1: TOF MS ES+
1.81e+004
226.0872
227.6768
m/z
For Help, press F1

20

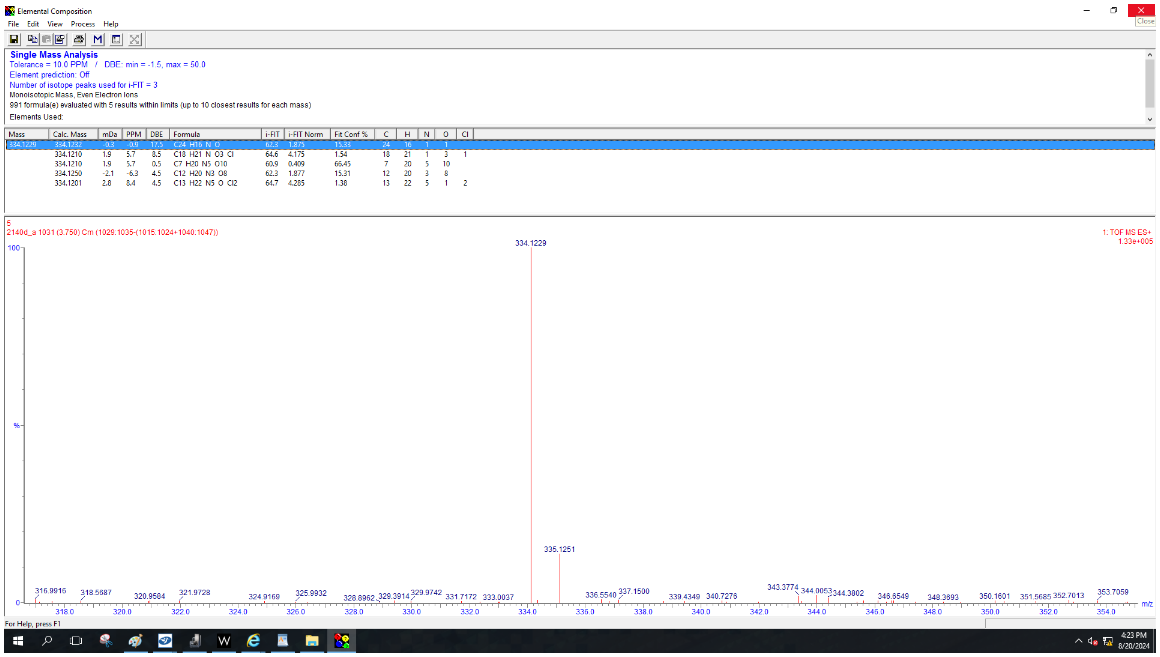

Elemental Composition

Single Mass Analysis
Tolerance = 10.0 PPM / DBE: min = -1.5, max = 50.0
Element prediction: Off
Number of isotope peaks used for i-FIT = 3
Monoisotopic Mass, Even Electron Ions
991 formula(e) evaluated with 5 results within limits (up to 10 closest results for each mass)
Elements Used:

Mass	Calc. Mass	mDa	PPM	DBE	Formula	i-FIT	i-FIT Norm	Fit Conf %	C	H	N	O	Cl
334.1229	334.1232	-0.3	-0.9	17.5	C24 H16 N O	62.3	1.875	15.33	24	16	1	1	
	334.1210	1.9	5.7	8.5	C18 H21 N O3 Cl	64.6	4.175	1.54	18	21	1	3	1
	334.1210	1.9	5.7	0.5	C7 H20 N5 O10	60.9	0.409	66.45	7	20	5	10	
	334.1250	-2.1	-6.3	4.5	C12 H20 N3 O8	62.3	1.877	15.31	12	20	3	8	
	334.1201	2.8	8.4	4.5	C13 H22 N5 O Cl2	64.7	4.285	1.38	13	22	5	1	2

21

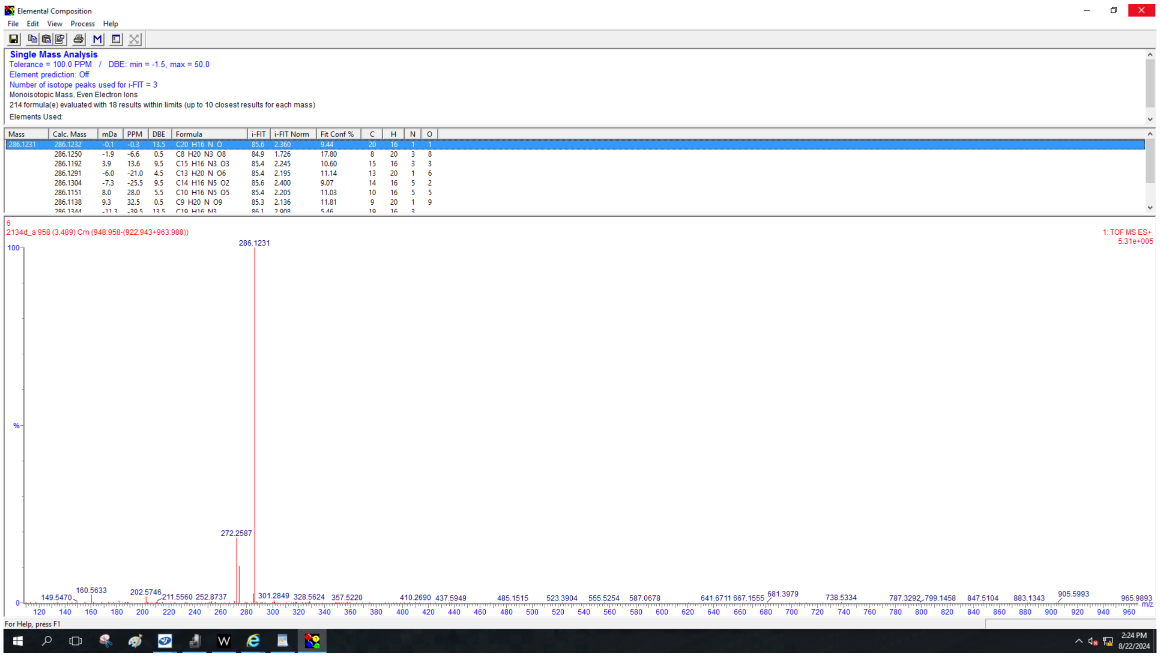

Elemental Composition

Single Mass Analysis
Tolerance = 100.0 PPM / DBE: min = -1.5, max = 50.0
Element prediction: Off
Number of isotope peaks used for i-FIT = 3
Monoisotopic Mass, Even Electron Ions
214 formula(e) evaluated with 18 results within limits (up to 10 closest results for each mass)
Elements Used:

Mass	Calc. Mass	mDa	PPM	DBE	Formula	i-FIT	i-FIT Norm	Fit Conf %	C	H	N	O
286.1231	286.1232	-0.1	-0.3	13.5	C20 H16 N O	85.6	2.360	9.44	20	16	1	1
	286.1250	-1.9	-6.6	0.5	C8 H20 N3 O8	84.9	1.726	17.80	8	20	3	8
	286.1192	3.9	13.6	9.5	C15 H16 N3 O3	85.4	2.245	10.60	15	16	3	3
	286.1291	-6.0	-21.0	4.5	C13 H20 N O6	85.4	2.195	11.14	13	20	1	6
	286.1304	-7.3	-25.5	9.5	C14 H16 N5 O2	85.6	2.400	9.07	14	16	5	2
	286.1151	8.0	28.0	5.5	C10 H16 N5 O5	85.4	2.205	11.03	10	16	5	5
	286.1138	9.3	32.5	0.5	C9 H20 N O9	85.3	2.136	11.81	9	20	1	9
	286.1344	-11.3	-39.5	13.5	C19 H16 N3	86.1	2.908	5.46	19	16	3	

23

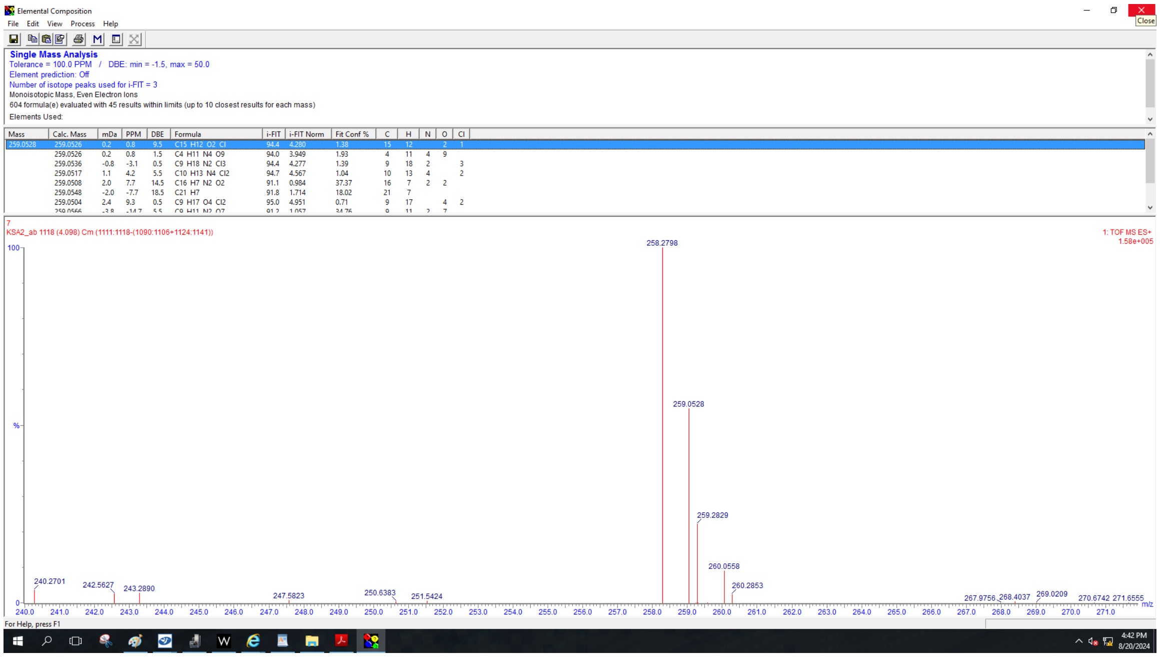
Elemental Composition
File Edit View Process Help
Single Mass Analysis
Tolerance = 100.0 PPM / DBE: min = -1.5, max = 50.0
Element prediction: Off
Number of isotope peaks used for i-FIT = 3
Monoisotopic Mass, Even Electron Ions
604 formula(e) evaluated with 45 results within limits (up to 10 closest results for each mass)
Elements Used:
Mass | Calc. Mass | mDa | PPM | DBE | Formula | i-FIT | i-FIT Norm | Fit Conf % | C | H | N | O | Cl
259.0528 259.0526 0.2 0.8 9.5 C15 H12 O2 Cl 94.4 4.280 1.38 15 12 2 1
259.0526 0.2 0.8 1.5 C4 H11 N4 O9 94.0 3.949 1.93 4 11 4 9
259.0536 -0.8 -3.1 0.5 C9 H18 N2 Cl3 94.4 4.277 1.39 9 18 2 3
259.0517 1.1 4.2 5.5 C10 H13 N4 Cl2 94.7 4.567 1.04 10 13 4 2
259.0508 2.0 7.7 14.5 C16 H7 N2 O2 91.1 0.984 37.37 16 7 2 2
259.0548 -2.0 -7.7 18.5 C21 H7 91.8 1.714 18.02 21 7
259.0504 2.4 9.3 0.5 C9 H17 O4 Cl2 95.0 4.951 0.71 9 17 4 2
7
KSA2_ab 1118 (4.098) Cm (1111:1118-(1090:1106+1124:1141))
1: TOF MS ES+
1.58e+005
258.2798
259.0528
259.2829
260.0558
260.2853
240.2701
242.5627
243.2890
247.5823
250.6383
251.5424
267.9756
268.4037
269.0209
270.6742
271.6555
m/z
For Help, press F1

24

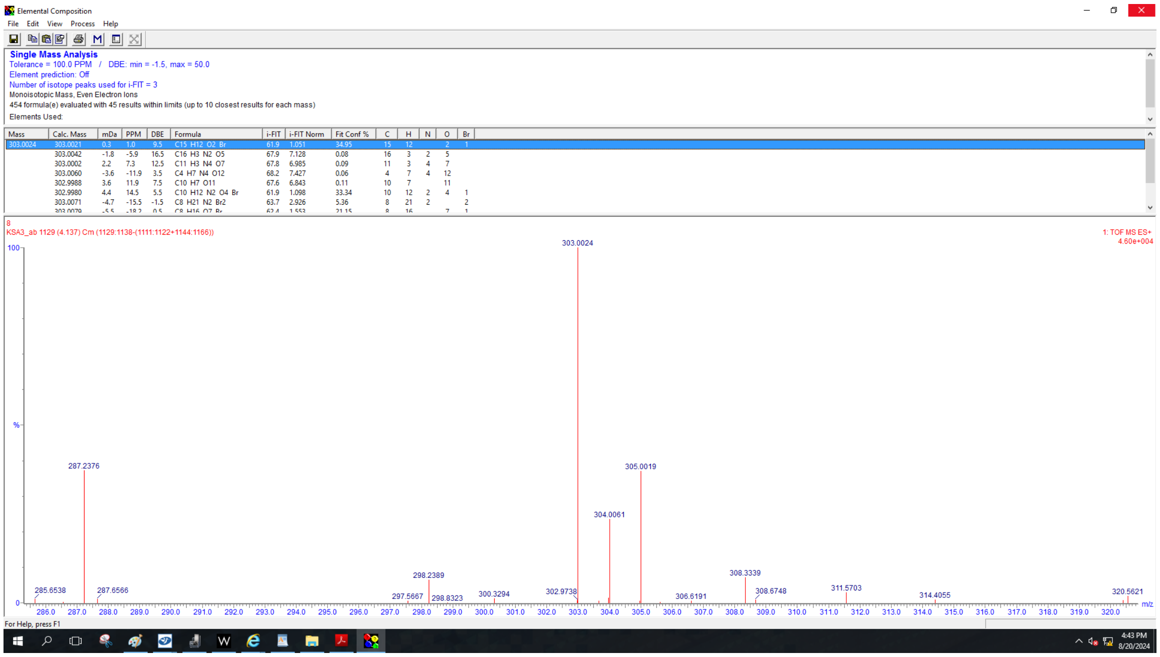
Elemental Composition
File Edit View Process Help
Single Mass Analysis
Tolerance = 100.0 PPM / DBE: min = -1.5, max = 50.0
Element prediction: Off
Number of isotope peaks used for i-FIT = 3
Monoisotopic Mass, Even Electron Ions
454 formula(e) evaluated with 45 results within limits (up to 10 closest results for each mass)
Elements Used:
Mass | Calc. Mass | mDa | PPM | DBE | Formula | i-FIT | i-FIT Norm | Fit Conf % | C | H | N | O | Br
303.0024 303.0021 0.3 1.0 9.5 C15 H12 O2 Br 61.9 1.051 34.95 15 12 2 1
303.0042 -1.8 -5.9 16.5 C16 H3 N2 O5 67.9 7.128 0.08 16 3 2 5
303.0002 2.2 7.3 12.5 C11 H3 N4 O7 67.8 6.985 0.09 11 3 4 7
303.0060 -3.6 -11.9 3.5 C4 H7 N4 O12 68.2 7.427 0.06 4 7 4 12
302.9988 3.6 11.9 7.5 C10 H7 O11 67.6 6.843 0.11 10 7 11
302.9980 4.4 14.5 5.5 C10 H12 N2 O4 Br 61.9 1.098 33.34 10 12 2 4 1
303.0071 -4.7 -15.5 -1.5 C8 H21 N2 Br2 63.7 2.926 5.36 8 21 2 2
8
KSA3_ab 1129 (4.137) Cm (1129:1138-(1111:1122+1144:1166))
1: TOF MS ES+
4.60e+004
303.0024
305.0019
304.0061
287.2376
285.6538
287.6566
297.5667
298.2389
298.8323
300.3294
302.9738
306.6191
308.3339
308.6748
311.5703
314.4055
320.5621
m/z
For Help, press F1

25

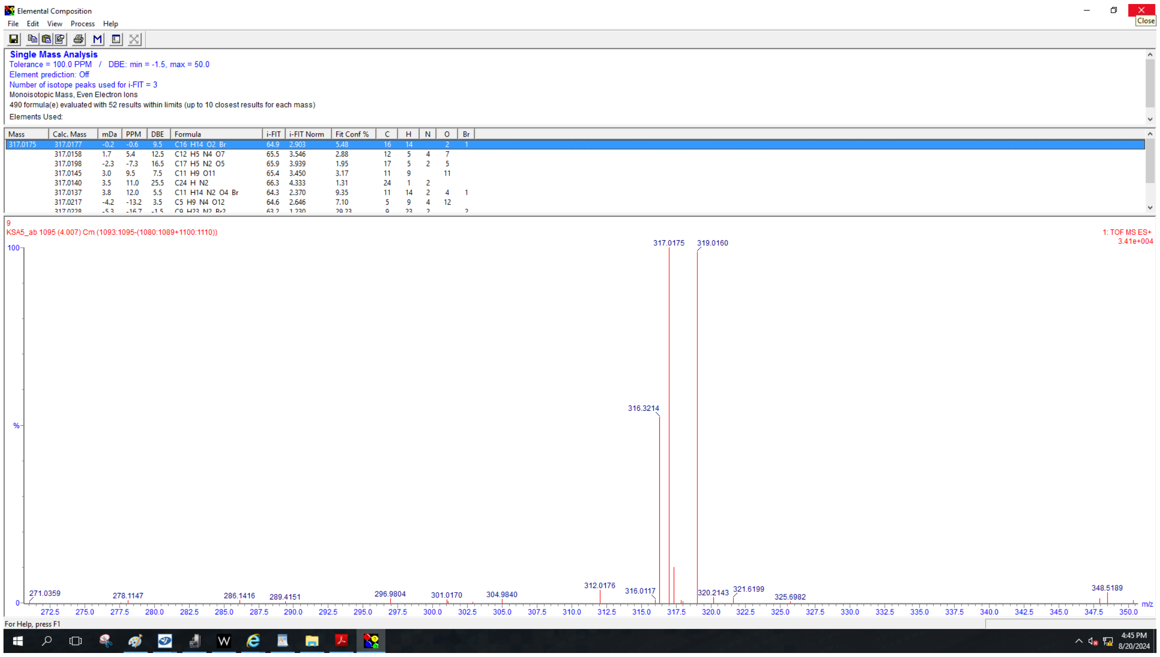
Elemental Composition
File Edit View Process Help
Single Mass Analysis
Tolerance = 100.0 PPM / DBE: min = -1.5, max = 50.0
Element prediction: Off
Number of isotope peaks used for i-FIT = 3
Monoisotopic Mass, Even Electron Ions
490 formula(e) evaluated with 52 results within limits (up to 10 closest results for each mass)
Elements Used:
KSA5_ab 1095 (4.007) Cm (1093:1095-(1080:1089+1100:1110))
1: TOF MS ES+
3.41e+004
317.0175
319.0160
316.3214
m/z
For Help, press F1

26

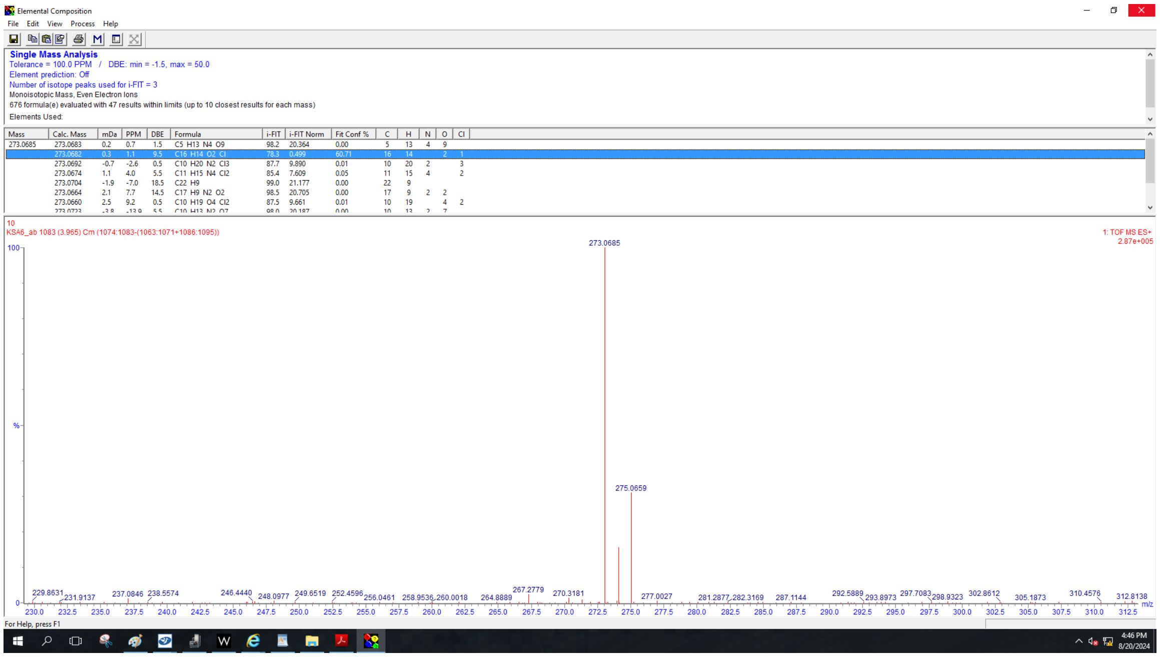
Elemental Composition
File Edit View Process Help
Single Mass Analysis
Tolerance = 100.0 PPM / DBE: min = -1.5, max = 50.0
Element prediction: Off
Number of isotope peaks used for i-FIT = 3
Monoisotopic Mass, Even Electron Ions
676 formula(e) evaluated with 47 results within limits (up to 10 closest results for each mass)
Elements Used:
KSA6_ab 1083 (3.965) Cm (1074:1083-(1063:1071+1086:1095))
1: TOF MS ES+
2.87e+005
273.0685
275.0659
m/z
For Help, press F1

27

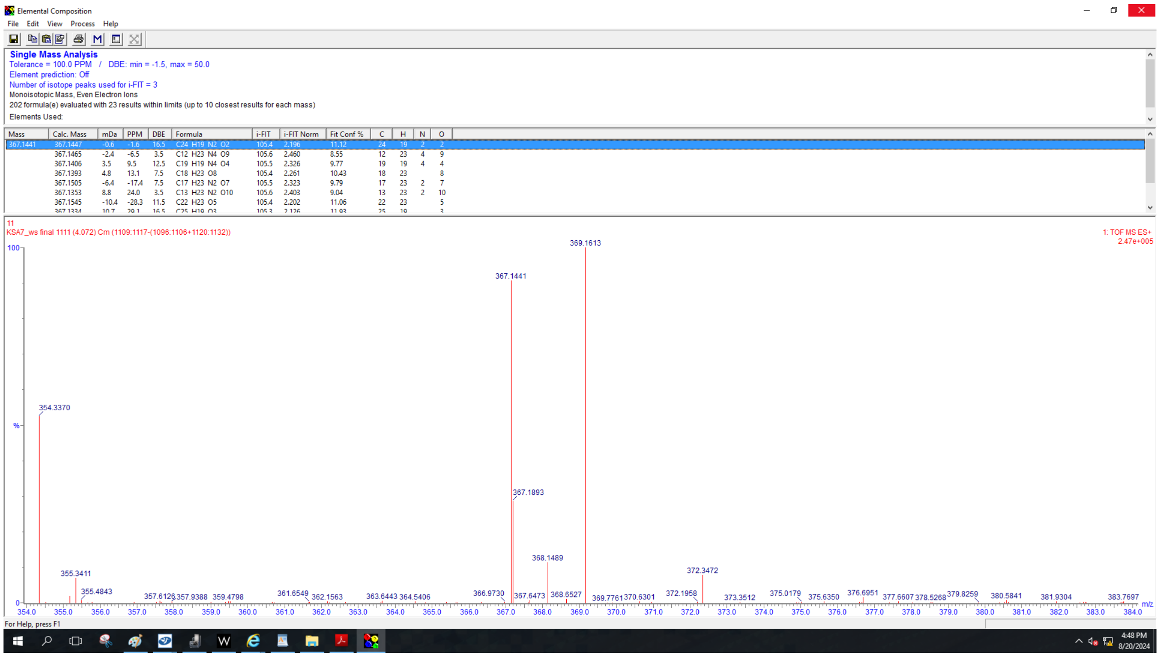
Elemental Composition
File Edit View Process Help
Single Mass Analysis
Tolerance = 100.0 PPM / DBE: min = -1.5, max = 50.0
Element prediction: Off
Number of isotope peaks used for i-FIT = 3
Monoisotopic Mass, Even Electron Ions
202 formula(e) evaluated with 23 results within limits (up to 10 closest results for each mass)
Elements Used:
Mass | Calc. Mass | mDa | PPM | DBE | Formula | i-FIT | i-FIT Norm | Fit Conf % | C | H | N | O
367.1441 | 367.1447 | -0.6 | -1.6 | 16.5 | C24 H19 N2 O2 | 105.4 | 2.196 | 11.12 | 24 | 19 | 2 | 2
367.1465 | -2.4 | -6.5 | 3.5 | C12 H23 N4 O9 | 105.6 | 2.460 | 8.55 | 12 | 23 | 4 | 9
367.1406 | 3.5 | 9.5 | 12.5 | C19 H19 N4 O4 | 105.5 | 2.326 | 9.77 | 19 | 19 | 4 | 4
367.1393 | 4.8 | 13.1 | 7.5 | C18 H23 O8 | 105.4 | 2.261 | 10.43 | 18 | 23 | 8
367.1505 | -6.4 | -17.4 | 7.5 | C17 H23 N2 O7 | 105.5 | 2.323 | 9.79 | 17 | 23 | 2 | 7
367.1353 | 8.8 | 24.0 | 3.5 | C13 H23 N2 O10 | 105.6 | 2.403 | 9.04 | 13 | 23 | 2 | 10
367.1545 | -10.4 | -28.3 | 11.5 | C22 H23 O5 | 105.4 | 2.202 | 11.06 | 22 | 23 | 5
11
KSA7_ws final 1111 (4.072) Cm (1109:1117-(1096:1106+1120:1132))
1: TOF MS ES+
2.47e+005
369.1613
367.1441
354.3370
367.1893
368.1489
372.3472
355.3411
For Help, press F1

28

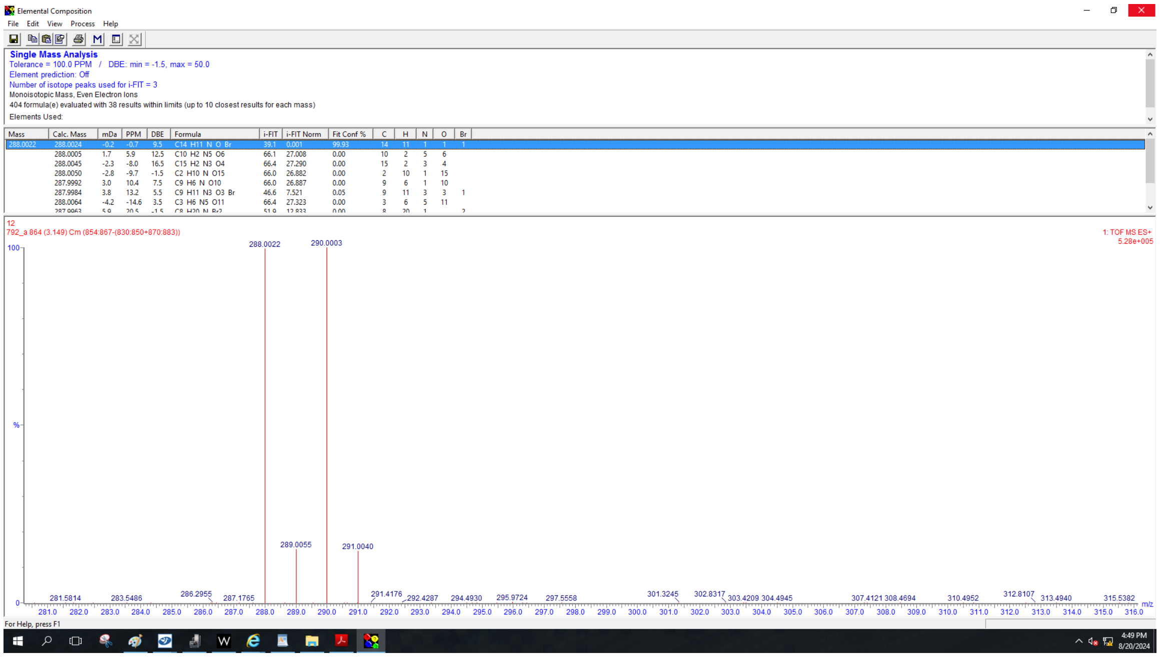
Elemental Composition
File Edit View Process Help
Single Mass Analysis
Tolerance = 100.0 PPM / DBE: min = -1.5, max = 50.0
Element prediction: Off
Number of isotope peaks used for i-FIT = 3
Monoisotopic Mass, Even Electron Ions
404 formula(e) evaluated with 38 results within limits (up to 10 closest results for each mass)
Elements Used:
Mass | Calc. Mass | mDa | PPM | DBE | Formula | i-FIT | i-FIT Norm | Fit Conf % | C | H | N | O | Br
288.0022 | 288.0024 | -0.2 | -0.7 | 9.5 | C14 H11 N O Br | 39.1 | 0.001 | 99.93 | 14 | 11 | 1 | 1 | 1
288.0005 | 1.7 | 5.9 | 12.5 | C10 H2 N5 O6 | 66.1 | 27.008 | 0.00 | 10 | 2 | 5 | 6
288.0045 | -2.3 | -8.0 | 16.5 | C15 H2 N3 O4 | 66.4 | 27.290 | 0.00 | 15 | 2 | 3 | 4
288.0050 | -2.8 | -9.7 | -1.5 | C2 H10 N O15 | 66.0 | 26.882 | 0.00 | 2 | 10 | 1 | 15
287.9992 | 3.0 | 10.4 | 7.5 | C9 H6 N O10 | 66.0 | 26.887 | 0.00 | 9 | 6 | 1 | 10
287.9984 | 3.8 | 13.2 | 5.5 | C9 H11 N3 O3 Br | 46.6 | 7.521 | 0.05 | 9 | 11 | 3 | 3 | 1
288.0064 | -4.2 | -14.6 | 3.5 | C3 H6 N5 O11 | 66.4 | 27.323 | 0.00 | 3 | 6 | 5 | 11
12
792_a 864 (3.149) Cm (854:867-(830:850+870:883))
1: TOF MS ES+
5.28e+005
288.0022
290.0003
289.0055
291.0040
For Help, press F1

29

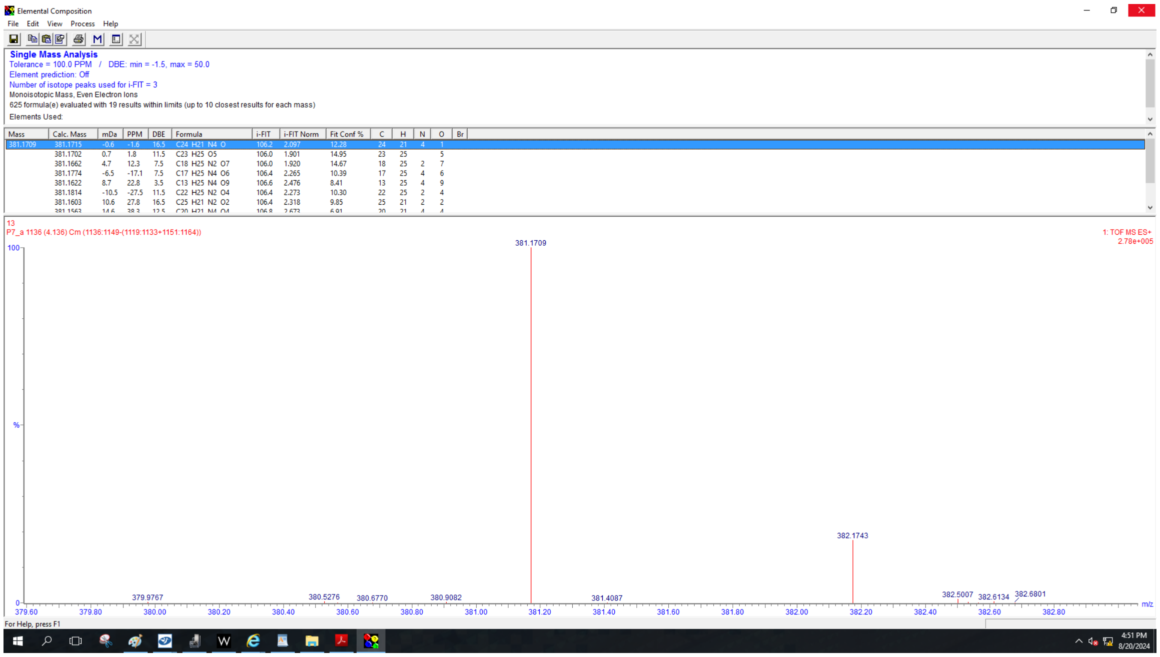

Mass	Calc. Mass	mDa	PPM	DBE	Formula	i-FIT	i-FIT Norm	Fit Conf %	C	H	N	O	Br
381.1709	381.1715	-0.6	-1.6	16.5	C24 H21 N4 O	106.2	2.097	12.28	24	21	4	1	
	381.1702	0.7	1.8	11.5	C23 H25 O5	106.0	1.901	14.95	23	25		5	
	381.1662	4.7	12.3	7.5	C18 H25 N2 O7	106.0	1.920	14.67	18	25	2	7	
	381.1774	-6.5	-17.1	7.5	C17 H25 N4 O6	106.4	2.265	10.39	17	25	4	6	
	381.1622	8.7	22.8	3.5	C13 H25 N4 O9	106.6	2.476	8.41	13	25	4	9	
	381.1814	-10.5	-27.5	11.5	C22 H25 N2 O4	106.4	2.273	10.30	22	25	2	4	
	381.1603	10.6	27.8	16.5	C25 H21 N2 O2	106.4	2.318	9.85	25	21	2	2	

30

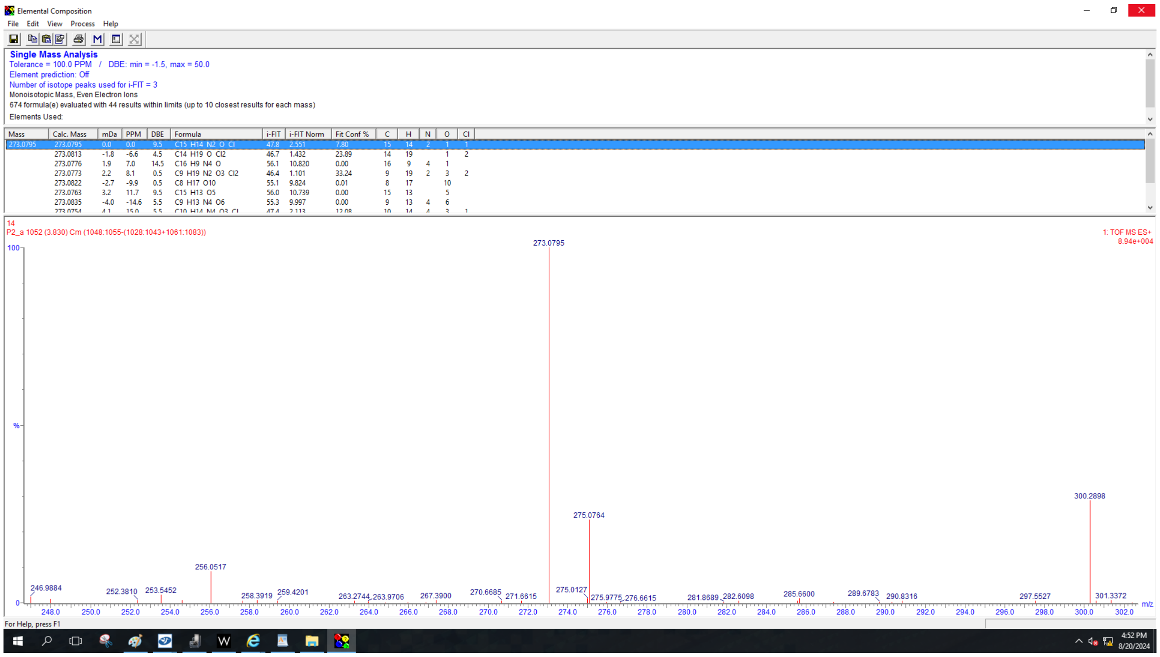

Mass	Calc. Mass	mDa	PPM	DBE	Formula	i-FIT	i-FIT Norm	Fit Conf %	C	H	N	O	Cl
273.0795	273.0795	0.0	0.0	9.5	C15 H14 N2 O Cl	47.8	2.551	7.80	15	14	2	1	1
	273.0813	-1.8	-6.6	4.5	C14 H19 O Cl2	46.7	1.432	23.89	14	19		1	2
	273.0776	1.9	7.0	14.5	C16 H9 N4 O	56.1	10.820	0.00	16	9	4	1	
	273.0773	2.2	8.1	0.5	C9 H19 N2 O3 Cl2	46.4	1.101	33.24	9	19	2	3	2
	273.0822	-2.7	-9.9	0.5	C8 H17 O10	55.1	9.824	0.01	8	17		10	
	273.0763	3.2	11.7	9.5	C15 H13 O5	56.0	10.739	0.00	15	13		5	
	273.0835	-4.0	-14.6	5.5	C9 H13 N4 O6	55.3	9.997	0.00	9	13	4	6	

31

Elemental Composition

File Edit View Process Help

Single Mass Analysis
Tolerance = 100.0 PPM / DBE: min = -1.5, max = 50.0
Element prediction: Off
Number of isotope peaks used for i-FIT = 3
Monoisotopic Mass, Even Electron Ions
489 formula(e) evaluated with 51 results within limits (up to 10 closest results for each mass)
Elements Used:

Mass	Calc. Mass	mDa	PPM	DBE	Formula	i-FIT	i-FIT Norm	Fit Conf %	C	H	N	O	Br
317.0292	317.0290	0.2	0.6	9.5	C15 H14 N2 O Br	37.8	1.434	23.84	15	14	2	1	1
	317.0297	-0.5	-1.6	11.5	C15 H9 O8	53.2	16.854	0.00	15	9		8	
	317.0311	-1.9	-6.0	16.5	C16 H5 N4 O4	53.4	17.048	0.00	16	5	4	4	
	317.0316	-2.4	-7.6	-1.5	C3 H13 N2 O15	52.9	16.492	0.00	3	13	2	15	
	317.0257	3.5	11.0	7.5	C10 H9 N2 O10	52.9	16.511	0.00	10	9	2	10	
	317.0249	4.3	13.6	5.5	C10 H14 N4 O3 Br	38.7	2.358	9.46	10	14	4	3	1
	317.0239	5.3	16.7	20.5	C22 H5 O3	54.0	17.633	0.00	22	5		3	
	317.0348	-5.6	-17.7	0.5	C8 H18 N2 O6 Br	38.2	1.775	16.94	8	18	2	6	1

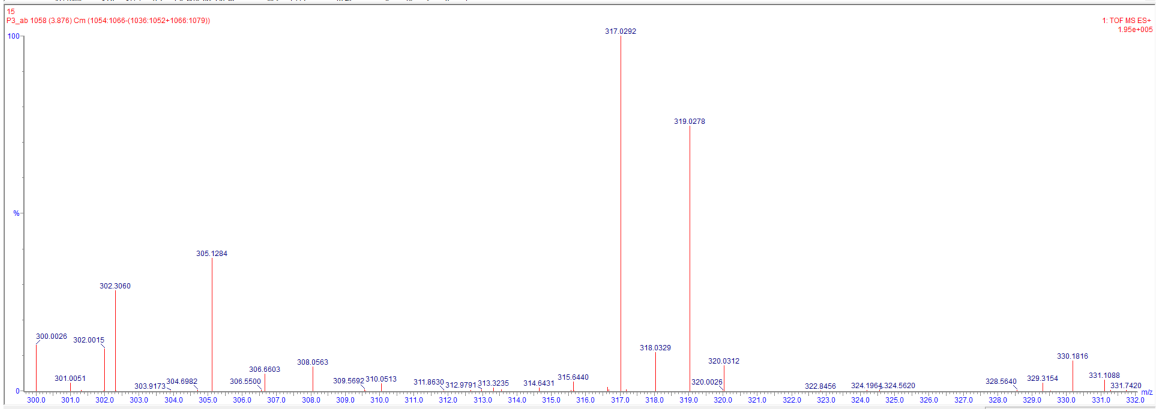

For Help, press F1
